# Components with Anti-Diabetic Activity Isolated from the Leaves and Twigs of *Glycosmis pentaphylla* Collected in Vietnam

**DOI:** 10.3390/ph15121543

**Published:** 2022-12-12

**Authors:** Minh Tuyet Thi Nguyen, I-Chi Hsu, Hui-Kang Liu, Yu-Chi Lin, Shu-Rong Chen, Fang-Rong Chang, Yuan-Bin Cheng

**Affiliations:** 1 Graduate Institute of Natural Products, College of Pharmacy, Kaohsiung Medical University, Kaohsiung 80708, Taiwan; 2 Division of Pharmacy, Zuoying Branch of Kaohsiung Armed Forces General Hospital, Kaohsiung 813204, Taiwan; 3 National Research Institute of Chinese Medicine, Ministry of Health and Welfare, Taipei 11221, Taiwan; 4 Department of Marine Biotechnology and Resources, National Sun Yat-sen University, Kaohsiung 80424, Taiwan

**Keywords:** *Glycosmis pentaphylla*, anti-diabetes, GLP-1, DPP4

## Abstract

A phytochemical investigation of the leaves and twigs of *Glycosmis pentaphylla* (Rutaceae), collected in Vietnam, yielded three new compounds named glyfuran (**1**), glyphyllamide (**2**), and glyphyllazole (**3**), along with twenty-five known compounds (**4**–**28**). The structures of isolates were determined by IR, MS, NMR, and UV data analyses. In the anti-diabetic activity screening, (+)-isoaltholacton (**4**), glycoborinine (**17**), 2′,4′-dihydroxy-4,6′-dimethoxychalcone (**24**), and flavokawain A (**25**) simultaneously exhibited inhibition of dipeptidyl peptidase-4 (DPP4) and stimulation of the glucagon-like peptide-1 (GLP-1) secretion on the murine intestinal secretin tumor cell line (STC-1).

## 1. Introduction

Diabetes mellitus is a metabolic disease characterized by excessive amounts of blood sugar, leading to a family of diseases that includes hypertension, osteoporosis, retinopathy, and urethritis. Along with the increasing popularity of a sedentary lifestyle, diabetes has gradually become a global epidemic. According to the World Health Organization, approximately 422 million people worldwide suffer from this disease, with up to 1.5 million dying yearly [1]. Owing to the eating habits of East Asians (rice is the staple food), the prevalence of diabetes is much higher than in other areas of the world. It is estimated that 8.5% of adults in Taiwan are afflicted with this disease, and the number of patients is predicted to increase in the future [2]. Anyway, the treatment of many diabetes complications is very costly and has become an economic burden for governments everywhere. Moreover, people with type 2 diabetes are twice as likely to develop liver or pancreatic cancer [3,4].

The leading causes of diabetes are the pancreas being unable to produce enough insulin (juvenile diabetes) or when the body becomes resistant to insulin (adult-onset diabetes). There is no cure for diabetes, but it can be treated and controlled, with treatment being divided into two kinds: subcutaneous insulin injection and oral hypoglycemic drugs. Oral hypoglycemic drugs induce effects that include the promotion of insulin secretion (Diamicron) with an increase in insulin receptor sensitivity (Metformin), α-glucosidase inhibitors (Acarbose), and dipeptidase-4 (DDP-4) inhibitors (Galvus). These medications often cause unpleasant side effects such as flatulence, diarrhea, hypoglycemia, weight gain, and even liver toxicity [1]. As a result, many people are turning to herbal medicines to treat diabetes [5]. So far, many Traditional Chinese Medicines or folk herbs have shown significant effects on lowering blood sugar or reducing the side effects of western medicines. For example, *Gastrodia elata* water extract can increase the quality of islet β cells and reduce cell apoptosis [6]; polyacetylene from *Bidens pilosa* var. *radiata* can decrease blood sugar and increase insulin release [7], while the polysaccharides in the flower buds of *Lonicera japonica* can lower blood sugar [8]. In terms of pure compounds, iminosugars and sugar derivatives were regarded as important antidiabetic agents [9,10,11,12,13].

Glucagon-like peptide-1 (GLP-1) is an incretin that can decrease blood sugar levels in a glucose-dependent manner by enhancing insulin secretion; moreover, the action of GLP-1 is preserved in patients with type 2 diabetes, and substantial pharmaceutical research has therefore been directed towards developing GLP-1-based treatment. However, endogenous GLP-1 is rapidly degraded primarily by dipeptidyl peptidase-4 (DPP-4) [14]. In our pre-screenings for anti-diabetes, the extract of *Glycosmis pentaphylla* (Retz.) DC. was able to simultaneously inhibit DPP-4 and stimulate the secretion of GLP-1 (Figure S1) in a dose-dependent manner (Figure S2), so *G. pentaphylla* has been proven to have the potential for development of an anti-diabetes drug and was accordingly selected for the following studies.

*G. pentaphylla*, a species of plant belonging to the family Rutaceae, is a shrub or small tree, 1.5–5.0 m high, and is widely distributed over India, Malaysia, Southern China, and the Philippine Islands to Vietnam. This plant is a Traditional Chinese Medicine (TCM) that can strengthen the stomach and relieve pain. In Vietnam, the roots, leaves, and branches of *G. pentaphylla* are collected year-round to relieve pain, to treat rheumatism, body aches, boils, impetigo, and snakebite. It is also used for postpartum women to cure uterine bleeding, eating indigestion, and abdominal distention [15,16]. Herein, the details of extraction, isolation, structural elucidation, anti-diabetes, and cytotoxicity of isolated compounds are described.

## 2. Results

**Figure 1:**
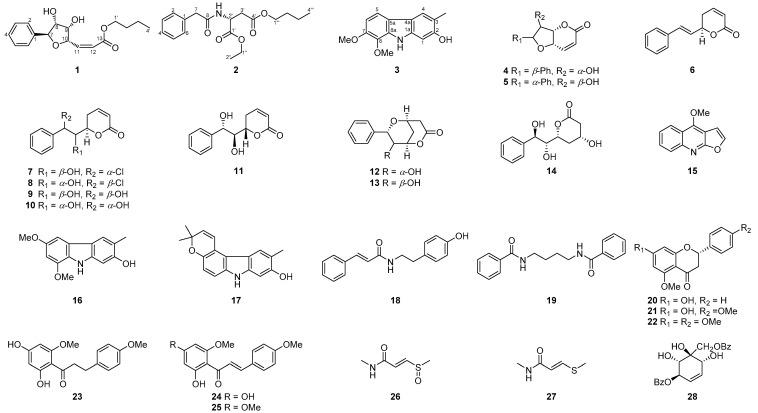
Structures of compounds **1**−**28**.

The air-dried twigs and leaves of *G. pentaphylla* were extracted with 95% ethanol. After partition and column chromatography, three new compounds named glyfuran (**1**), glyphyllamide (**2**), and glyphyllazole (**3**), along with 25 known compounds, (+)-isoaltholactone (**4**) [17], (+)-altholactone (**5**) [18], 6*R*-goniothalamin (**6**) [19], (6*R*,7*R*,8*S*)-8-chlorogoniodiol (**7**) [20], (6*R*,7*S*,8*R*)-8-chlorogoniodiol (**8**) [21], 7-*epi*-(+)-gonidiol (**9**) [22], 8-*epi*-(+)-goniodiol (**10**) [23], (6*S*,7*S*,8*S*)-goniodiol (**11**) [24], (+)-9-deoxygoniopypyrone (**12**) [25], (−)-8-*epi*-9-deoxygoniopyrone (**13**) [25], leiocarpin C (**14**) [26], dictamine (**15**) [27], 2-hydroxy-6,8-dimethoxy-3-methyl-9*H*-carbazole (**16**) [28], glycoborinine (**17**) [29], *N*-(4-hydroxyphenethyl)cinnamamide (**18**) [30], uvariadiamide (**19**) [31], alpinetin (**20**) [32], tsugafolin (**21**) [33], naringenin trimethyl ether (**22**) [33], 4′,6′-dihyroxy-2′,4-dimethoxydihydrochalcone (**23**) [34], 2′,4′-dihyroxy-4,6′-dimethoxychalcone (**24**) [35], flavokawain A (**25**) [36], glycothiomin-A (**26**) [37], penangin (**27**) [38], and ellipeiopsol B (**28**) [39], were obtained (Figure 1).

Compound **1** was observed as a white amorphous powder with ${\left[\alpha \right]}_{D}^{24}$ + 54 (*c* 0.05, MeOH). The molecular formula C_17_H_22_O_5_ (seven indices of hydrogen deficiency) of **1** was deduced from a sodium adduct peak at *m*/*z* 329.13605 in the HRESIMS. The IR spectrum indicated the presence of hydroxy (3380 cm^−1^) and ester carbonyl (1684 cm^−1^) groups. The ^1^H NMR data (Table 1) of **1** clearly demonstrated the presence of one mono-substituted benzene ring [*δ*_H_ 7.29 (m, H-4), 7.36 (2H, m, H-3/H-5), 7.39 (2H, m, H-2/H-6)], two olefinic protons [*δ*_H_ 6.00 (dd, *J* = 11.8, 1.8 Hz, H-12), *δ*_H_ 6.46 (dd, *J* = 11.8, 6.5 Hz, H-11)], four oxymethines [*δ*_H_ 4.16 (t, *J* = 5.0 Hz, H-8), *δ*_H_ 4.60 (t, *J* = 5.0 Hz, H-9), 5.00 (d, *J* = 5.6 Hz, H-7), and 5.65 (ddd, *J* = 6.5, 5.6, 1.8 Hz, H-10)], and one methyl group [*δ*_H_ 0.95 (t, *J* = 7.5 Hz, H-4′)]. The ^13^C NMR and DEPT spectra (Table 1) exhibited seventeen carbons, containing one ester group (*δ*_C_ 167.2), one olefinic quaternary carbon (*δ*_C_ 140.2), seven olefinic methines (*δ*_C_ 120.9, 125.6, 125.6, 127.8, 128.5, 128.5, and 148.2), four oxymethines (*δ*_C_ 73.7, 78.7, 79.0, and 84.0), one oxymethylene (*δ*_C_ 64.8), two methylenes (*δ*_C_ 19.1 and 30.6), and one methyl group (*δ*_C_ 13.6).

**Figure 2:**
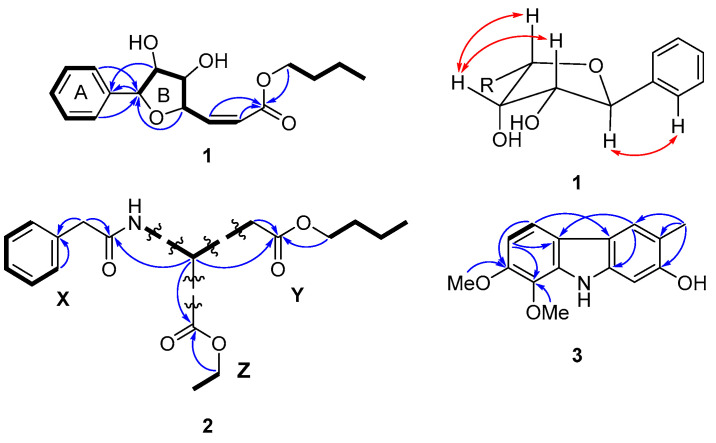
Key COSY (bold), HMBC (arrow), and NOESY (double arrow) correlations of **1**–**3**.

In the COSY spectrum (Figure 2) of **1**, three proton sequences of H-2/H-3/H-4/H-5/H-6, H-7/H-8/H-9/H-10/H-11/H-12, and H_2_-1′ (*δ*_H_ 4.15)/H_2_-2′ (*δ*_H_ 1.66)/H_2_-3′ (*δ*_H_ 1.40)/H_3_-4′ (*δ*_H_ 0.95) were observed. The proton spin-spin coupling systems of H-2/H-3/H-4/H-5/H-6 and the HMBC correlations (Figure 2) of H-7 to C-1 (*δ*_C_ 140.2) and H-2/H-6 to C-7 (*δ*_C_ 84.0) revealed the presence of a monosubstituted benzene ring (ring A), while the sequences of H-7/H-8/H-9/H-10 and the HMBC correlation from H-10 to C-7 (*δ*_C_ 84.0) suggested **1** contained a tetrahydrofuran ring (ring B). The *n*-butyl ester attached at C-12 was constructed by the COSY consequences H_2_-1′/H_2_-2′/H_2_-3′/H_3_-4′ and the HMBC correlation from H-11, H-12, and H-1′ to C-13 (*δ*_C_ 167.2). On the basis of these definitive 2D NMR analyses, the planar structure of **1** was established.

Moreover, in the NOESY spectrum of **1**, the presence of NOESY correlations (Figure 2) between H-11 and H-12 validated the *cis* conformation of Δ_11_. The NOESY cross-peaks of H-8/H-9/H-10 suggested they were *β*-orientated; on the other hand, the NOESY correlations of H-1/H-7 revealed these protons were *α*-orientated.

**Figure 3:**
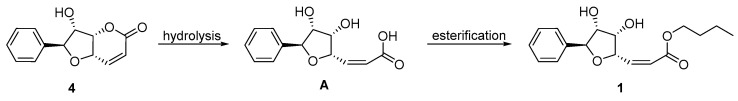
The plausible biosynthetic pathway of **1** from **4**.

Additionally, compound **4** is the major component of *G. Pentaphylla* (0.006%), suggesting compound **1** might be derived from **4** (Figure 3). After hydrolysis, **4** might become intermediate **A**, and **A** was esterificated to form **1**. Through comparison between compounds **1** and **4**, the absolute configuration of **1** was established and assigned the trivial name glyfuran.

Compound **2** was isolated as a white amorphous powder with $\;{\left[\alpha \right]}_{D}^{24}$ − 10 (*c* 0.05, MeOH). The high-resolution ESIMS data showed a protonated molecule peak at [M + H]^+^ at *m*/*z* 336.18067, which indicated the molecular formula of C_18_H_25_NO_5_ and seven degrees of unsaturation. The IR spectrum of **2** showed absorption bands at 1736 and 1671 cm^−1^, revealing the presence of ester and amide functionality. The ^1^H NMR data (Table 2) of **2** possessed the signals of one monosubstituted benzene ring [*δ*_H_ 7.29 (2H, m, H-3/H-5), 7.31 (m, H-4), and 7.37 (2H, m, H-2/H-6)], two methylenes [*δ*_H_ 4.02 (q, *J* = 7.1 Hz, H-1‴) and *δ*_H_ 4.19 (q, *J* = 7.1 Hz, H-1″)], and two methyl groups [*δ*_H_ 0.93 (t, *J* = 7.4 Hz, H-4‴) and *δ*_H_ 1.24 (t, *J* = 7.1 Hz, H-2″)]. The ^13^C NMR and DEPT spectra exhibited eighteen carbons, including two ester carbonyls (*δ*_C_ 170.5 and 170.8), one amide carbonyl (*δ*_C_ 170.7), one olefinic quaternary carbon (*δ*_C_ 134.4), five olefinic methines (*δ*_C_ 127.4, 128.9, 128.9, 129.3, and 129.3), two oxymethylenes (*δ*_C_ 61.8 and 64.9), one *N*-bearing methine (*δ*_C_ 48.6), four methylenes (*δ*_C_ 19.0, 36.2, 30.4, and 43.6), and two methyl groups (*δ*_C_ 13.6 and 14.0). The planar structure of **2** was elucidated by dividing it into three partial structures, X, Y, and Z (Figure 2). A proton spin system of H-2 (*δ*_H_ 7.37)/H-3 (*δ*_H_ 7.29)/H-4 (*δ*_H_ 7.31)/H-5 (*δ*_H_ 7.29)/H-6 (*δ*_H_ 7.37) was observed from the COSY spectrum. In the HMBC spectrum, H_2_-7 (*δ*_H_ 3.62) showed correlations to C-1 (*δ*_C_ 134.4) and C-8 (*δ*_C_ 170.7), and the correlation from H-2/H-6 to C-1 revealed the presence of partial structure X. For the partial structure Y, a butyl group attached to an ester group was identified by the COSY correlations of H-1‴ (*δ*_H_ 4.02)/H-2‴ (*δ*_H_ 1.54)/H-3‴ (*δ*_H_ 1.33)/H-4‴ (*δ*_H_ 0.93) and the HMBC correlations from H_2_-3′ (*δ*_H_ 2.82 and 3.00) and H-1‴ to C-4′ (*δ*_C_ 170.8). Additionally, the HMBC correlations from H-1″ (*δ*_H_ 4.19) to C-1′ (*δ*_C_ 170.5), along with the proton spin system between H-1″ (*δ*_H_ 4.19) and H-2″ (*δ*_H_ 1.24) established the partial structure Z. These three parts were connected by the HMBC correlations from H-2′ (*δ*_H_ 4.83) to C-8, C-1′, and C-4′. According to the above 2D NMR analyses, the planar structure of **2** was established (Figure 2). Structurally, **2** showed similar ^1^H and ^13^C NMR data to a synthetic compound *N*-PhAc-_D_-Asp(OEt)OEt [40]. In addition, the negative optical rotation values of **2** (−10) and *N*-PhAc-_D_-Asp(OEt)OEt (−39) suggested the *R* configuration of C-2′.

Compound **3** was isolated as a brown-yellow solid and had a molecular formula of C_15_H_15_NO_3_ based on HRESIMS data (*m*/*z* 280.09466 [M + Na]^+^), accounting for nine degrees of unsaturation. The IR spectrum showed the absorption bands due to hydroxy (3412 cm^−1^), amide (1625 cm^−1^), and phenyl (1512 and 1443 cm^−1^) functional groups. The UV spectrum displayed absorption maxima at 307 and 259 nm; therefore, **3** was defined to have a carbazole skeleton [41]. The ^1^H NMR spectrum (Table 3) of **3** showed the presence of three singlet protons, one for a phenolic hydroxy group at *δ*_H_ 7.94 (brs, 2-OH) and the other two for aromatic methines at *δ*_H_ 6.83 (s, H-1) and 7.67 (s, H-4), as well as a couple of aromatic protons [*δ*_H_ 6.84 (d, *J* = 8.5 Hz, H-6) and 7.56 (d, *J* = 8.5 Hz, H-5)]. The ^1^H NMR spectrum also displayed a methyl at *δ*_H_ 2.39 (s, 3-Me), along with two methoxy groups at *δ*_H_ 3.96 (s, 7-OMe) and *δ*_H_ 4.00 (s, 8-OMe). The ^13^C NMR exhibited the signals of fifteen carbons, being twelve aromatic carbons (*δ*_C_ 96.8, 106.2, 114.3, 116.2, 117.8, 119.5, 121.4, 133.7, 134.0, 139.6, 149.4, and 152.5), one methyl carbon (*δ*_C_ 16.1), and two methoxy carbon groups (*δ*_C_ 56.9 and 60.9). The COSY correlation showed one fragment of H-5/H-6. In the HMBC experiment, ^3^*J* correlations from *δ*_H_ 3.96 to *δ*_C_ 149.4 (C-7) and from *δ*_H_ 4.00 to *δ*_C_ 133.7 (C-8) indicated the positions of two methoxy groups. In addition, the correlations from 3-Me to C-2 (*δ*_C_ 152.5), C-3 (*δ*_C_ 116.2), and C-4 (*δ*_C_ 121.4) revealed the position of a methyl group. The HMBC correlations from H-4 to C-5a (*δ*_C_ 119.5)/C-1a (*δ*_C_ 139.6) and from H-5 to C-4a (*δ*_C_ 117.8) were used to construct the pyrrole moiety. Therefore, compound **3** was determined to be 7,8-dimethoxy-3-methyl-9*H*-carbazol-2-ol, and the trivial name glyphyllazole was given.

**Figure 4:**
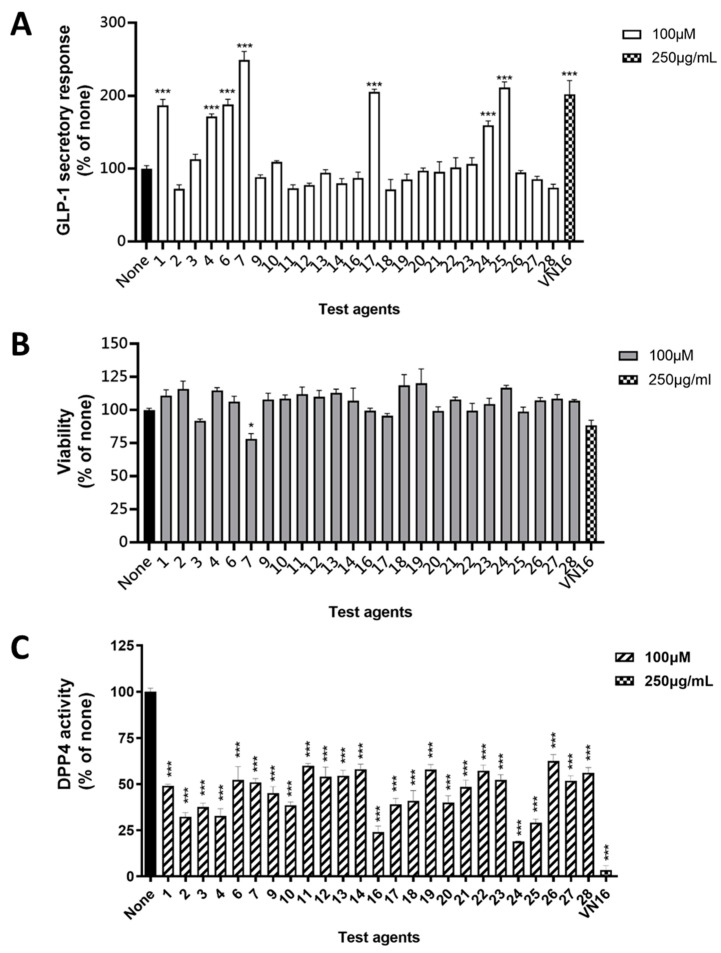
The anti-diabetes effect of isolated compounds in STC-1 cells. (**A**) GLP-1 secretory effects of the methanol layer of *G. pentaphylla* (VN16) and isolated compounds. (**B**) Acute toxicity evaluation of the methanol layer of *G. pentaphylla* (VN16) and isolated compounds in STC-1 cells. (**C**) The effects of the methanol layer of *G. pentaphylla* (VN16) and isolated compounds on DPP4. Data represent mean ± SEM (*n* = 3–4). Ordinary one-way ANOVA with Dunnett’s multiple comparisons test. * *p* < 0.05, *** *p* < 0.001 when compared to None.

The anti-diabetic activity of isolated secondary metabolites was evaluated by stimulating the secretion of GLP-1 and inhibiting DPP-4. As shown in Figure 4A, compounds **1**, **4**, **6**, **7**, **17**, **24**, and **25** exhibited stimulatory effects on GLP-1 secretion from murine intestinal secretin tumor cell line (STC-1) at a concentration of 100 μM. In the acute toxicity evaluation (Figure 4B), except for compound **7**, all tested compounds retained the cell viability of STC-1 cells at the concentration of 100 μM. On the other hand, all tested compounds provided inhibitory effects on DPP-4 enzyme activities (Figure 4C); significantly, compounds **2**–**4**, **9**, **10**, **16**–**18**, **20**, **24**, and **25** demonstrated inhibition rates over 50%.

## 3. Materials and Methods

### 3.1. General Experimental Procedures

The optical rotations, UV, and IR spectra were recorded on a Jasco P-2000 digital polarimeter, a Jasco V-530 UV/VIS spectrophotometer, and a Jasco FT-IR 4600 spectrometer, respectively. NMR spectra data were corrected in CDCl_3_ (*δ*_H_ 7.26 and *δ*_C_ 77.0) using solvent peaks as the internal standard on Bruker AVIII HD 700X NMR and Varian VNMRS 600 MHz spectrometers. HRESIMS data were obtained by using a Bruker 7T solariX spectrometer. Column chromatography was performed using Merck silica gel 60 (0.040–0.063 mm), C_18_ silica gel (0.040–0.075 mm), and Sephadex LH-20 material. NP-HPLC was composed of a Hitachi L-6000 pump and a Hitachi L-4000 UV detector with Phenomenex Luna silica (5 μm, 250 × 10 mm) and CN (5 μm, 250 × 10 mm) columns. RP-HPLC was carried out on a Shimadzu chromatography system consisting of a LC-20AT pump, a SPD-M20A PDA detector, and a CBM-20A system controller with Phenomenex Luna C_18_ (5 μm, 250 × 10 mm), phenyl-hexyl (5 μm, 250 × 10 mm), and Kinetex biphenyl (5 μm, 250 × 10 mm) columns.

### 3.2. Plant Material

The leaves and twigs of *G. pentaphylla* were purchased in July 2018 from Cu Lao Cham Island (Hoi An City, Quang Nam province, Vietnam) by Associate Professor Quang Vinh Nguyen (Institute of Biotechnology and Environment, Tay Nguyen University, Vietnam) and Associate Professor Chia-Hung Yen (Graduate Institute of Natural Products, Kaohsiung Medical University, Taiwan). A voucher specimen (code No. KMU-VN016) was deposited at Kaohsiung Medical University.

### 3.3. Extraction and Isolation

The dried leaves and twigs of *G. pentaphylla* (0.7 kg) were extracted by 95% EtOH (5 L × 3, each for 3 days) at room temperature to give an ethanolic extract, which was then partitioned between EtOAc and H_2_O (1:1) to provide an EtOAc layer. The EtOAc layer was further partitioned by hexanes/MeOH/H_2_O (4:3:1) to obtain a 75% MeOH_(aq)_ layer (13.3 g). This methanol extract was subjected to a silica gel flash column (hexanes/CH_2_Cl_2_/MeOH, 60/10/1 to 0/0/1) to afford subfractions GP-1 to GP-6. GP-3 (5.2 g) was separated by a silica gel open column stepwise-eluted with hexanes/EtOAc (10/1 to 0/1) and EtOAc/MeOH (5/1 to 0/1) to yield 18 subfractions (GP-3-1 to GP-3-18). GP-3-7 (80.5 mg) was subjected to a Sephadex LH-20 open column eluting with CH_2_Cl_2_/MeOH (1/1) to obtain four fractions (GP-3-7-1 to GP-3-7-4). Compound **25** (2.1 mg) was purified from GP-3-7-4 (17.8 mg) by semi-preparative RP-HPLC (phenyl-hexyl column, flow = 2.0 mL/min, 65% MeCN_(aq)_, isocratic, 360 nm). GP-3-10 (342.6 mg) was applied to chromatography on a silica gel open column (hexanes/CH_2_Cl_2_/MeOH, 1/1/0 to 0/0/1) to give **6** (32.5 mg) and fractions GP-3-10-2 to GP-3-10-4. GP-3-10-3 (220.9 mg) was further separated on a Sephadex LH-20 column (CH_2_Cl_2_/MeOH, 1/1) to afford seven fractions (GP-3-10-3-1 to GP-3-10-3-7). GP-3-10-3-5 (12.4 mg) was isolated by semi-preparative NP-HPLC (silica column, flow = 2.0 mL/min, hexanes/CH_2_Cl_2_/MeOH = 60/10/1, isocratic, 280 nm) to obtain **2** (3.1 mg). Compounds **1** (2.5 mg), **15** (1.1 mg), and **23** (38.1 mg) were purified from GP-3-10-3-6 (108.4 mg) by repeated semi-preparative NP-HPLC (silica and CN columns, flow = 2.0 mL/min, hexanes/CH_2_Cl_2_/MeOH = 70/10/1, isocratic, 280 nm). GP-3-11 (1.8 g) was fractionated by a Sephadex LH-20 column eluted with CH_2_Cl_2_/MeOH (1/1) to give five fractions (GP-3-11-1 to GP-3-11-5). GP-3-11-3 (167.3 mg) was subjected to semi-preparative RP-HPLC (C_18_ column, flow = 2.0 mL/min, 30% MeCN_(aq)_, isocratic, 280 nm) to afford **4** (38.6 mg), **5** (4.0 mg), **7** (12.9 mg), **8** (1.5 mg), and **18** (1.4 mg). GP-3-11-4 (237.2 mg) was isolated by semi-preparative RP-HPLC (phenyl-hexyl column, flow = 2.0 mL/min, 50% MeCN_(aq)_, isocratic, 280 nm) to yield **21** (1.4 mg) and **24** (43.2 mg). GP-3-11-5 (23.1 mg) was purified by semi-preparative RP-HPLC (C_18_ column, flow = 2.0 mL/min, 50% MeCN_(aq)_, isocratic, 280 nm) to obtain **3** (3.5 mg), **16** (1.9 mg), and **17** (2.9 mg). GP-3-12 (315.4 mg) was subjected to Sephadex LH-20 column eluted with CH_2_Cl_2_/MeOH (1/1) to obtain five fractions (GP-3-12-1 to GP-3-12-5). Compound **22** (6.2 mg) was purified from GP-3-12-3 (55.8 mg) by semi-preparative RP-HPLC (C_18_ column, flow = 2.0 mL/min, 60% MeCN_(aq)_, isocratic, 280 nm). Compound **28** (1.7 mg) was isolated from GP-3-12-4 (84.1 mg) by semi-preparative RP-HPLC (phenyl-hexyl column, flow = 2.0 mL/min, 30% MeCN_(aq)_, isocratic, 210 and 280 nm). GP-3-13 (218.5 mg) was processed using a Sephadex LH-20 open column eluted with CH_2_Cl_2_/MeOH (1/1). GP-3-13-2 (153.3 mg) was fractionated over C_18_ open column (H_2_O/MeOH, 100/0 to 0/100) to give GP-3-13-2-1 to GP-3-13-2-5. Compounds **11** (15.3 mg) and **13** (4.0 mg) were obtained from GP-3-13-2-1 (24.1 mg) by semi-preparative RP-HPLC (phenyl-hexyl column, flow = 2.0 mL/min, 20% MeCN_(aq)_, isocratic, 254 nm). Compounds **10** (12.7 mg) and **14** (13.5 mg) were isolated from GP-3-13-2-2 (65.2 mg) by repeated semi-preparative RP-HPLC (phenyl-hexyl and biphenyl columns, flow = 2.0 mL/min, 25% MeCN_(aq)_, isocratic, 254 nm). GP-3-13-2-3 (17.5 mg) was purified by semi-preparative RP-HPLC (phenyl-hexyl column, flow = 2.0 mL/min, 40% MeOH_(aq)_, isocratic, 254 nm) to yield **9** (4.7 mg). GP-3-13-2-4 (13.7 mg) was isolated by semi-preparative RP-HPLC (phenyl-hexyl column, flow = 2.5 mL/min, 60% MeOH_(aq)_, isocratic, 280 nm) to afford **20** (3.2 mg). GP-3-14 (282.1 mg) was subjected to Sephadex LH-20 (CH_2_Cl_2_/MeOH, 1/1) and ODS (H_2_O/MeOH, 100/0 to 0/100) open columns, respectively. Compound **12** (2.9 mg) was purified from GP-3-14-2-2 (18.8 mg) by semi-preparative RP-HPLC (C_18_ column, flow = 2.0 mL/min, 30% MeCN_(aq)_, isocratic, 254 nm). GP-3-15 (364.1 mg) was chromatographed by a Sephadex LH-20 open column eluted with CH_2_Cl_2_/MeOH (1/1) to obtain three fractions (GP-3-15-1 to GP-3-15-3). GP-3-15-3 (163.5 mg) was applied to a C_18_ gel open column (H_2_O/MeOH, 100/0 to 0/100) to obtain fractions GP-3-15-3-1 to GP-3-15-3-5. Compounds **26** (18.8 mg) and **27** (44.8 mg) were purified by semi-preparative RP-HPLC (biphenyl column, flow = 2.0 mL/min, 10% MeCN_(aq)_, isocratic, 280 nm). GP-3-15-3-3 (18.6 mg) was isolated by semi-preparative RP-HPLC (C_18_ column, flow = 2.0 mL/min, 30% MeCN_(aq)_, isocratic, 280 nm) to give **19** (8.8 mg).

*Glyfuran* (**1**)*:* White amorphous powder; ${\left[\alpha \right]}_{D}^{24}$ + 54 (*c* 0.05, MeOH); UV (MeOH) *λ*_max_ (log *ε*) 276 (3.23), 226 (3.20) nm; IR (KBr) *v*_max_ 3380, 2923, 2853, 1684, 1456, 1259, 1192, 1026 cm^−1^; ^1^H NMR and ^13^C NMR data, see Table 1; HRESIMS *m*/*z* 329.13605 [M + Na]^+^ (calcd for C_17_H_22_NaO_5_, 329.13594).

*Glyphyllamide* (**2**)*:* White amorphous powder; ${\left[\alpha \right]}_{D}^{24}$ − 10 (*c* 0.05, MeOH); UV (MeOH) *λ*_max_ (log *ε*) 285 (2.83), 213 (3.53) nm; IR (KBr) *v*_max_ 2925, 2854, 1736, 1671, 1457, 1179 cm^−1^; ^1^H NMR and ^13^C NMR data, see Table 2; HRESIMS *m*/*z* 336.18067 [M + H]^+^ (calcd for C_18_H_26_NO_5_, 336.18055).

*Glyphyllazole* (**3**)*:* Brown-yellow solid; UV (MeOH) *λ*_max_ (log *ε*) 307 (3.80), 259 (3.96), 237 (2.23), 212 (4.0) nm; IR (KBr) *v*_max_ 3142 (OH), 2925, 1626, 1513, 1443, 1296, 1229, 1140, 1068 cm^−1^; ^1^H NMR and ^13^C NMR data, see Table 3; HRESIMS *m*/*z* 280.09468 [M + Na]^+^ (calcd for C_15_H_15_NNaO_3_, 280.09441).

### 3.4. Anti-Diabetic Assays

#### 3.4.1. Cell Culture

Murine enteroendocrine cell line (STC-1) purchased from American Type Culture Collection (ATCC) was maintained in Dulbecco’s modified Eagle’s medium (DMEM) containing 15% (*v*/*v*) horse serum (HS) and 5% (*v*/*v*) fetal bovine serum (FBS).

#### 3.4.2. GLP-1 Secretion

Cells were seeded into a 24-well plate at 1.5 × 10^5^ cells/well. After 72 h, cells were washed with glucose-free DMEM at 0.1% BSA three times and then replaced with DMEM (5.5 mM glucose) with/without VN016 or testing compound preparations for 1 h. At the end of treatment, the supernatant was collected and measured for GLP-1 using an active GLP-1 assay kit (Cisbio). The viability of cells after extract or compound treatment was measured by neutral red assay according to the previous description [42].

#### 3.4.3. DPP-IV Activity Assay

According to the manufacturer’s instructions, the measurement of the activity and potential inhibition of DPP-IV, a type II membrane glycoprotein, was performed using the DPP-IV GloTM Protease Assay (Promega).

## 4. Conclusions

As part of an ongoing program to search for bioactive compounds from Vietnam’s medicinal plants, a chemical study on the leaves and twigs of *G. pentaphylla* has been carried out, resulting in the purification of three new (**1**−**3**) and twenty-five known (**4**–**28**) compounds. This research led to the isolation of eleven styryl-lactones (**4**–**14**) and five alkaloids (**15**–**19**), which proved the genus *Glycosmis* is a rich source of both alkaloid and styryl-lactone. In anti-diabetic evaluation, compounds **4**, **17**, **24**, and **25** displayed dual activities by stimulating GLP-1 secretion while inhibiting DPP-4 (which can rapidly degrade GLP-1) without cytotoxicity in STC-1 cells. Consequently, our pharmacological data supports *G. pentaphylla* as being a folk medicine with a hypoglycemic effect.

## Figures and Tables

**Table 1:** ^1^H NMR (600 MHz) and ^13^C NMR (150 MHz) spectroscopic data of **1** in CDCl_3_.

No.	1	
	*δ*_H_ (Mult, *J* in Hz)	*δ*_C_, Type
1	–	140.2, C
2	7.39, m	125.6, CH
3	7.36, m	128.5, CH
4	7.29, m	127.8, CH
5	7.36, m	128.5, CH
6	7.39, m	125.6, CH
7	5.00, d (5.6)	84.0, CH
8	4.16, t (5.0)	78.7, CH
9	4.60, t (5.0)	73.7, CH
10	5.65, ddd (6.5, 5.6, 1.8)	79.0, CH
11	6.46, dd (11.8, 6.5)	148.2, CH
12	6.00, dd (11.8, 1.8)	120.9, CH
13	–	167.2, C
1′	4.15, t (5.0)	64.8, CH_2_
2′	1.66, m	30.6, CH_2_
3′	1.40, m	19.1, CH_2_
4′	0.95, t (7.5)	13.6, CH_3_

**Table 2:** ^1^H NMR (700 MHz) and ^13^C NMR (175 MHz) spectroscopic data of **2** in CDCl_3_.

No.	2	
	*δ*_H_ (Mult, *J* in Hz)	*δ*_C_, Type
1	–	134.4, C
2	7.37, m	128.9, CH
3	7.29, m	129.3, CH
4	7.31, m	127.4, CH
5	7.29, m	129.3, CH
6	7.37, m	128.9, CH
7	3.62, s	43.6, CH2
8	–	170.7, C
1′	–	170.5, C
2′	4.83, m	48.6, CH
3′	2.82, dd (17.0, 4.6)	36.2, CH2
	3.00, dd (17.0, 4.3)	
4′	–	170.8, C
1″	4.19, q (7.1)	61.8, CH2
2″	1.24, t (7.1)	14.0, CH3
1‴	4.02, t (7.1)	64.9, CH2
2‴	1.54, m	30.4, CH2
3‴	1.33, m	19.0, CH2
4‴	0.93, t (7.4)	13.6, CH3
NH	6.46, d (7.6)	–

**Table 3:** ^1^H NMR (600 MHz) and ^13^C NMR (150 MHz) spectroscopic data of **3** in CDCl_3_.

No.	3	
	*δ*_H_ (Mult, *J* in Hz)	*δ*_C_, Type
1	6.83, s	96.8, CH
1a	–	139.6, C
2	–	152.5, C
3	–	116.2, C
4	7.67, s	121.4, CH
4a	–	117.8, C
5	7.56, d (8.5)	114.3, CH
5a	–	119.5, C
6	6.84, d (8.5)	106.2, CH
7	–	149.4, C
8	–	133.7, C
8a	–	134.0, C
2-OH	7.94, br s	–
3-Me	2.39, s	16.1, CH_3_
7-OMe	3.96, s	56.9, CH_3_
8-OMe	4.00, s	60.9, CH_3_

## Data Availability

Data is contained within the article and in the Supplementary Materials.

## Supplementary Materials

The following supporting information can be downloaded at: https://www.mdpi.com/article/10.3390/ph15121543/s1, Figure S1: Preliminary screening of dual biological activities related to modulation of GLP-1 levels; Figure S2: dose-dependent effects of VN016-MeOH; Figures S3–S27: HR-ESI-MS, IR, NMR (^1^H, ^13^C, COSY, HSQC, HMBC, and NOESY), and UV spectra of compounds **1**–**3**.
