# Supplementary material for: Components with Anti-Diabetic Activity Isolated from the Leaves and Twigs of Glycosmis pentaphylla Collected in Vietnam
Source: Pharmaceuticals (Basel). 2022 Dec 12;15(12):1543. doi: 10.3390/ph15121543 (PMC9785221; doi:10.3390/ph15121543)

Supporting Information

Components with Anti-Diabetic Activity Isolated from the Leaves and Twigs of *Glycosmis pentaphylla* Collected in Vietnam

Minh Tuyet Thi Nguyen [1,†], I-Chi Hsu [2,†], Hui-Kang Liu [3], Yu-Chi Lin [3], Shu-Rong Chen [4], Fang-Rong Chang [1], Yuan-Bin Cheng [4,*]

[1] Graduate Institute of Natural Products, College of Pharmacy, Kaohsiung Medical University, Kaohsiung 80708, Taiwan
[2] Division of Pharmacy, Zuoying Branch of Kaohsiung Armed Forces General Hospital, Kaohsiung 813204, Taiwan
[3] National Research Institute of Chinese Medicine, Ministry of Health and Welfare, Taipei 11221, Taiwan
[4] Department of Marine Biotechnology and Resources, National Sun Yat-sen University, Kaohsiung 80424, Taiwan
* Correspondence: jmb@mail.nsysu.edu.tw; Tel.: +886-7-5252000 ext. 5212
† These authors contributed equally to this work.

Table of Contents

Figure S1. Preliminary screening of dual biological activities related to modulation of GLP-1 levels....3

Figure S2. Dose dependent effects of VN016-MeOH....3

Figure S3. ^{1}H NMR spectrum of **1** ($CDCl_3$, 600 MHz)....4

Figure S4. ^{13}C NMR and DEPT spectra of **1** ($CDCl_3$, 150 MHz)....5

Figure S5. COSY spectrum of **1**....6

Figure S6. HSQC spectrum of **1**....7

Figure S7. HMBC spectrum of **1**....8

Figure S8. NOESY spectrum of **1**....9

Figure S9. HRESIMS spectrum of **1**....10

Figure S10. UV spectrum of **1**....11

Figure S11. IR spectrum of **1**....11

Figure S12. ^{1}H NMR spectrum of **2** ($CDCl_3$, 700 MHz)....12

Figure S13. ^{13}C NMR and DEPT spectra of **2** ($CDCl_3$, 175 MHz)....13

Figure S14. COSY spectrum of **2**....14

Figure S15. HSQC spectrum of **2**....15

Figure S16. HMBC spectrum of **2**....16

Figure S17. HRESIMS spectrum of **2**....17

Figure S18. UV spectrum of **2**....18

Figure S19. IR spectrum of **2**....18

Figure S20. ^{1}H NMR spectrum of **3** ($CDCl_3$, 600 MHz)....19

Figure S21. ^{13}C NMR and DEPT spectra of **3** ($CDCl_3$, 150 MHz)....20

Figure S22. COSY spectrum of **3**....21

Figure S23. HSQC spectrum of **3**....22

Figure S24. HMBC spectrum of **3**....23

Figure S25. HRESIMS spectrum of **3**....24

Figure S26. UV spectrum of **3**....25

Figure S27. IR spectrum of **3**....25

Figure S1. Preliminary screening of dual biological activities related to modulation of GLP-1 levels. (A) DPP-IV activity and (B) GLP-1 secretion were employed to screen the four layers of *Glycosmis pentaphylla* (VN016) with dual activities. Data represents Mean ± SEM (n = 2–3). *P<0.05 and **P<0.01 when compared with None.

(A) **(B)**

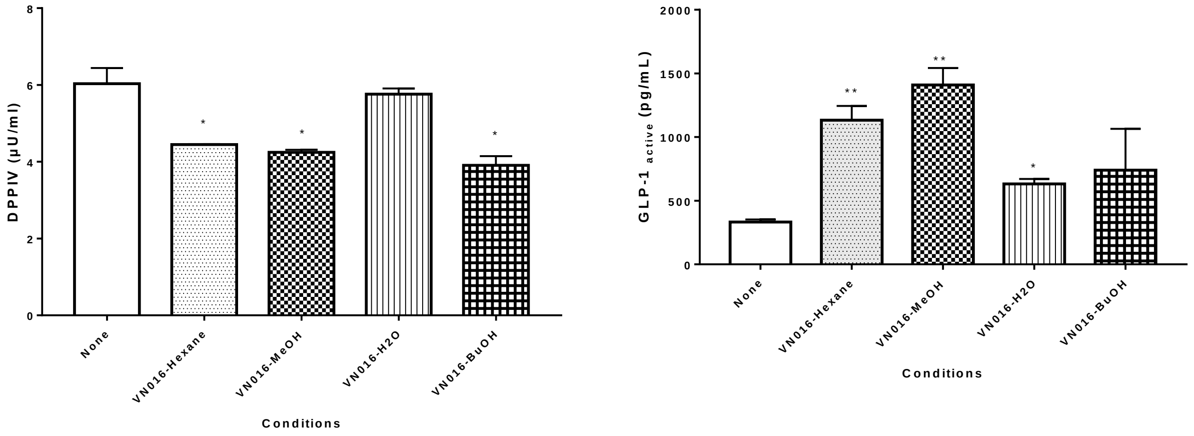

Figure S2. Dose dependent effects of *G. pentaphylla* (VN016-MeOH). (A) DPP-IV activity and (B) GLP-1 secretion were employed to test dose-dependent effects of VN016-MeOH with dual activities. Data represents Mean ± SEM (n = 2–3). *P<0.05 and **P<0.01 when compared with None.

(A) **(B)**

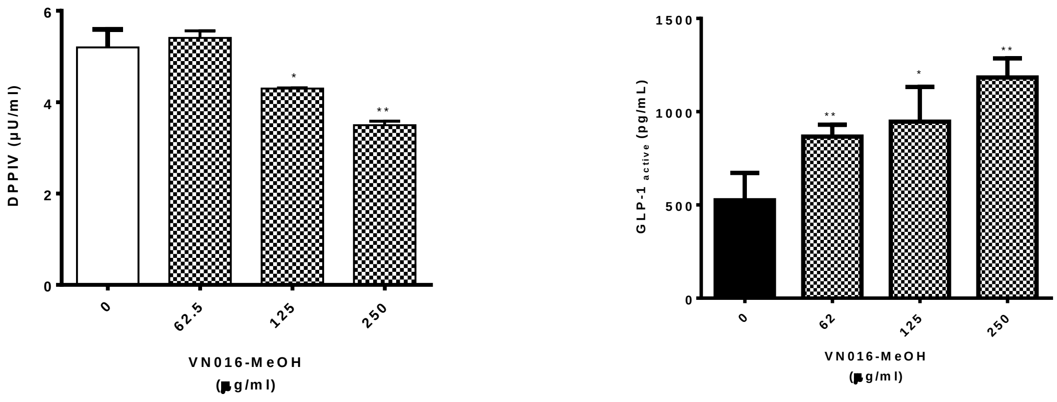

Figure S3. ^{1}H NMR spectrum of **1** ($CDCl_3$, 600 MHz)

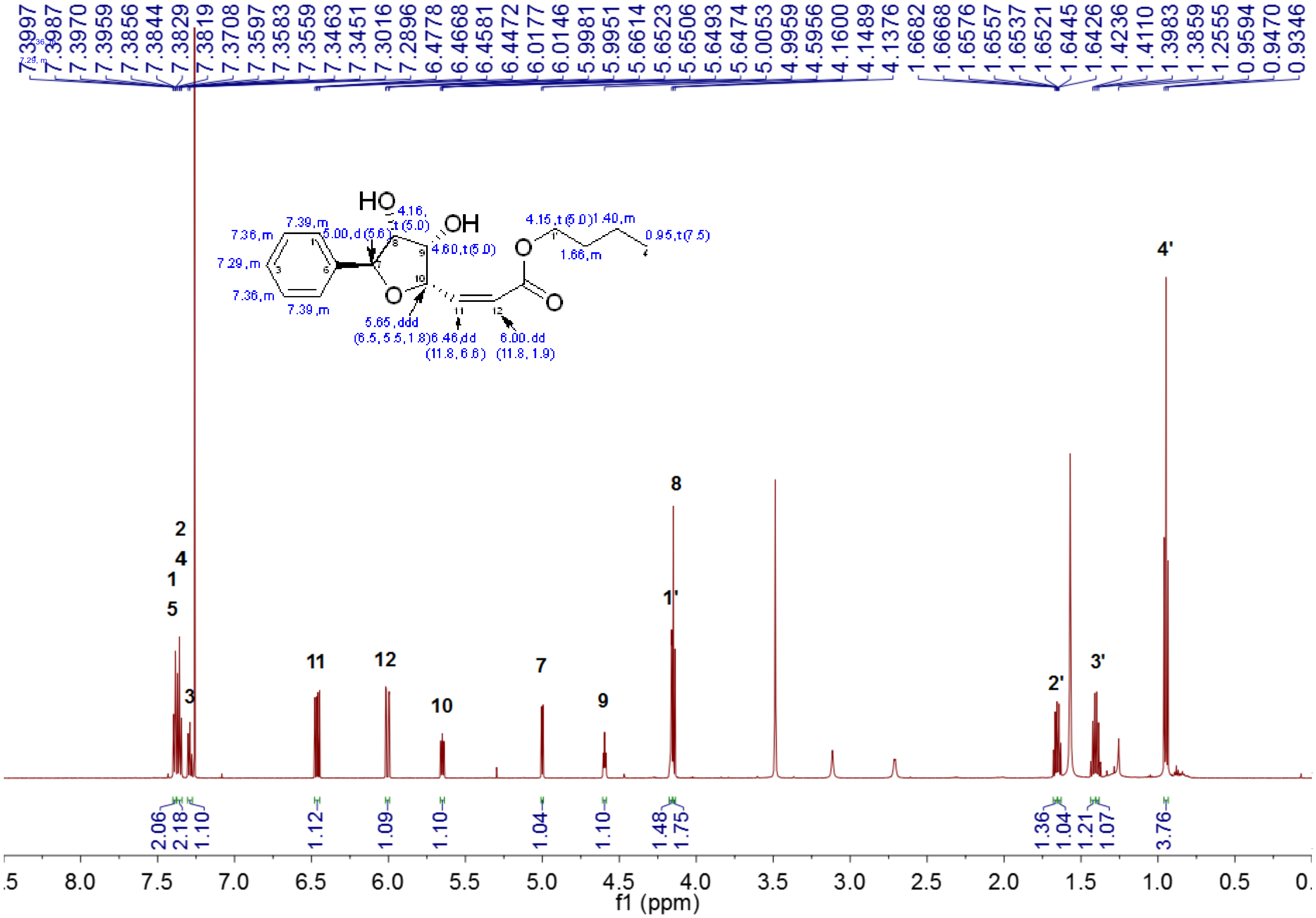

Figure S4. ^{13}C NMR and DEPT spectra of **1** ($CDCl_3$, 150 MHz)

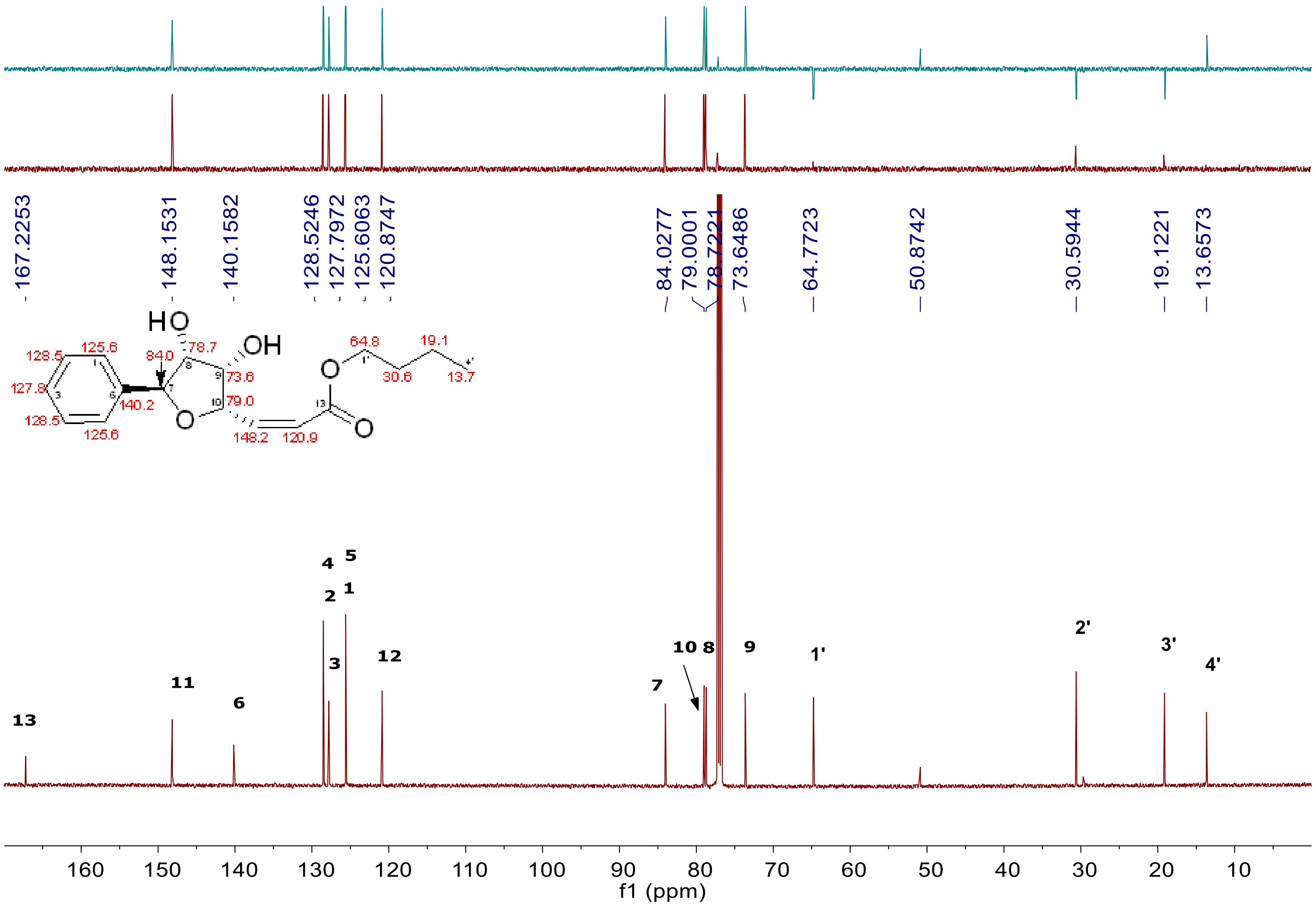

Figure S5. COSY spectrum of **1**

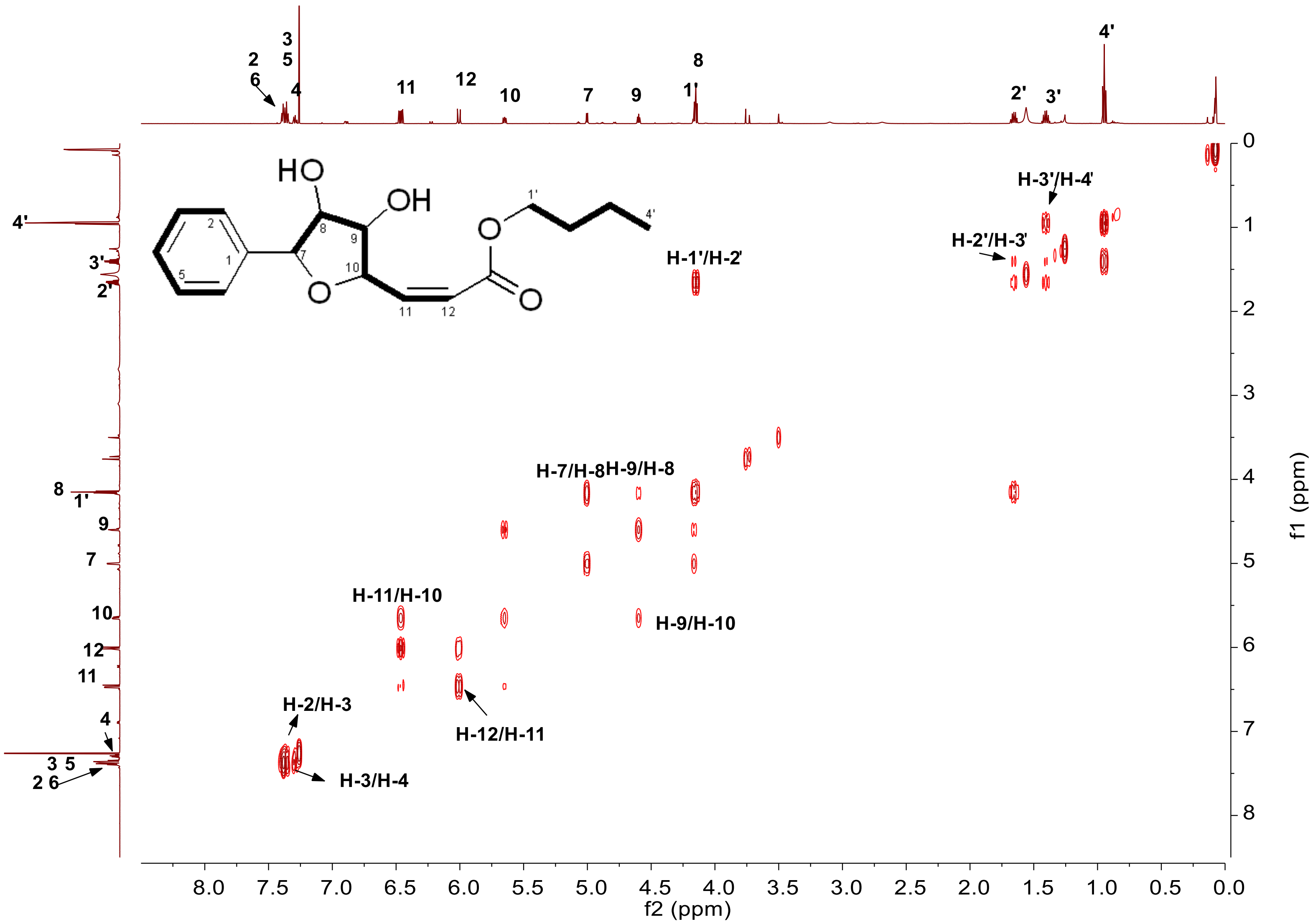

Figure S6. HSQC spectrum of **1**

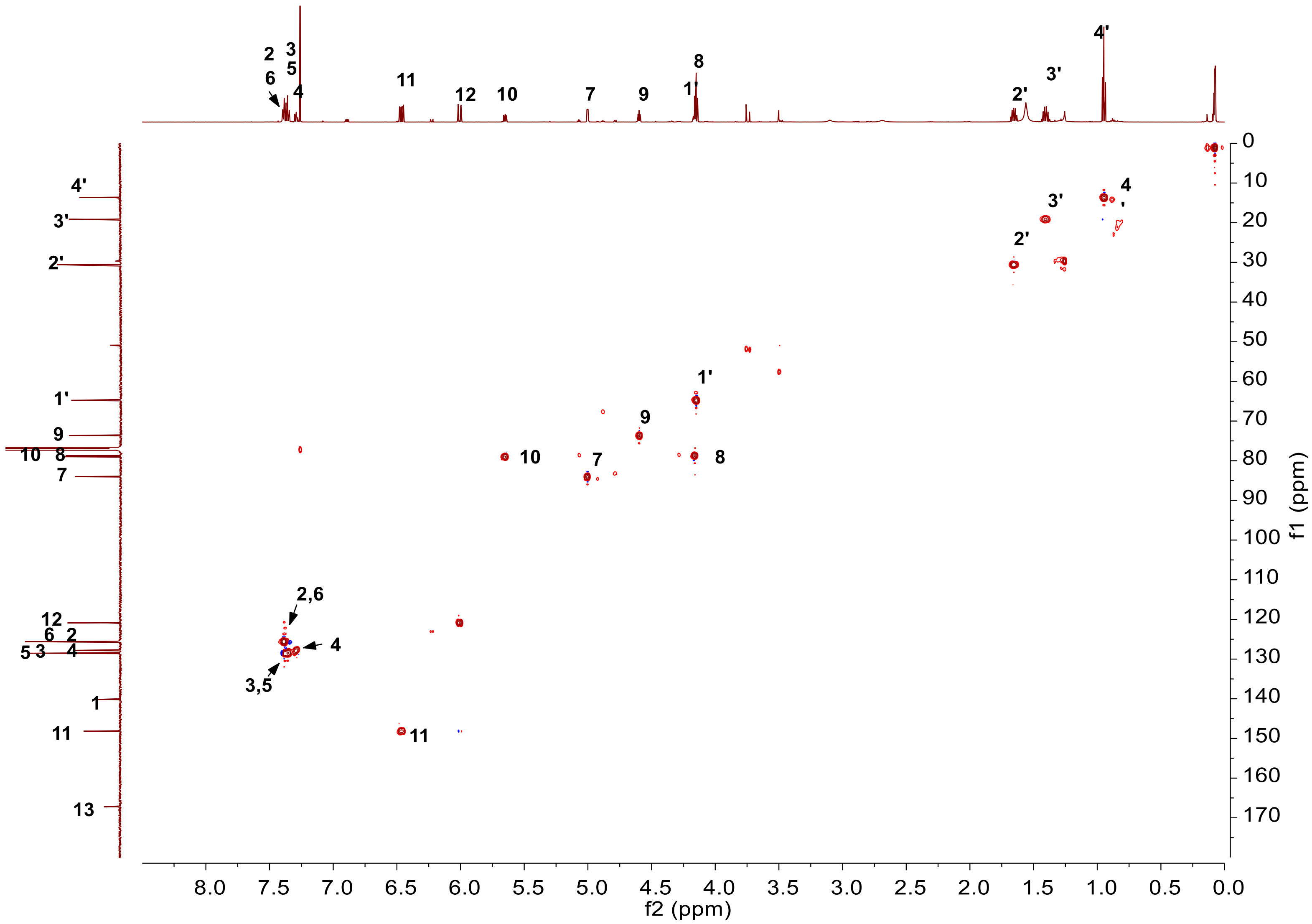

Figure S7. HMBC spectrum of **1**

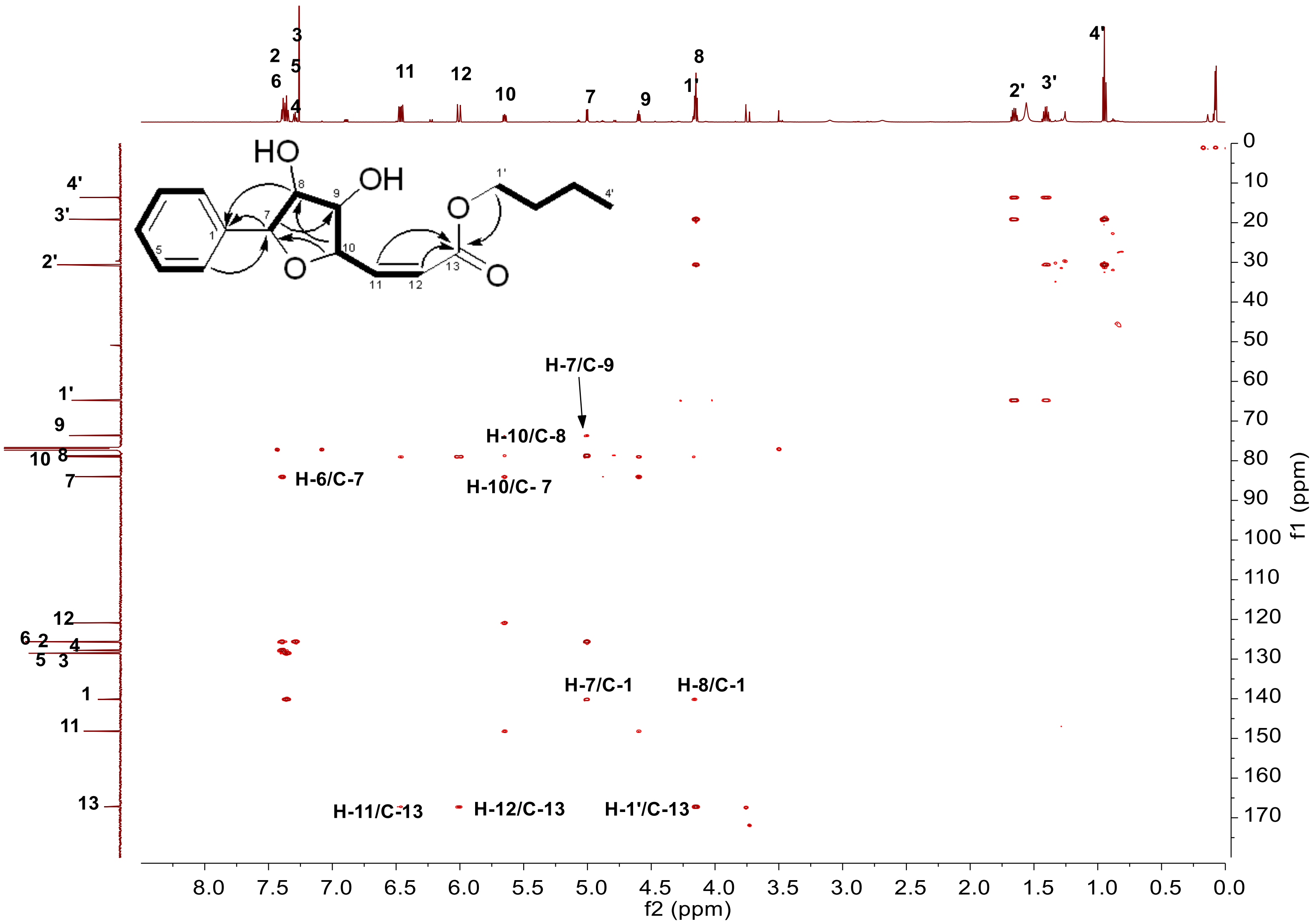

Figure S8. NOESY spectrum of **1**

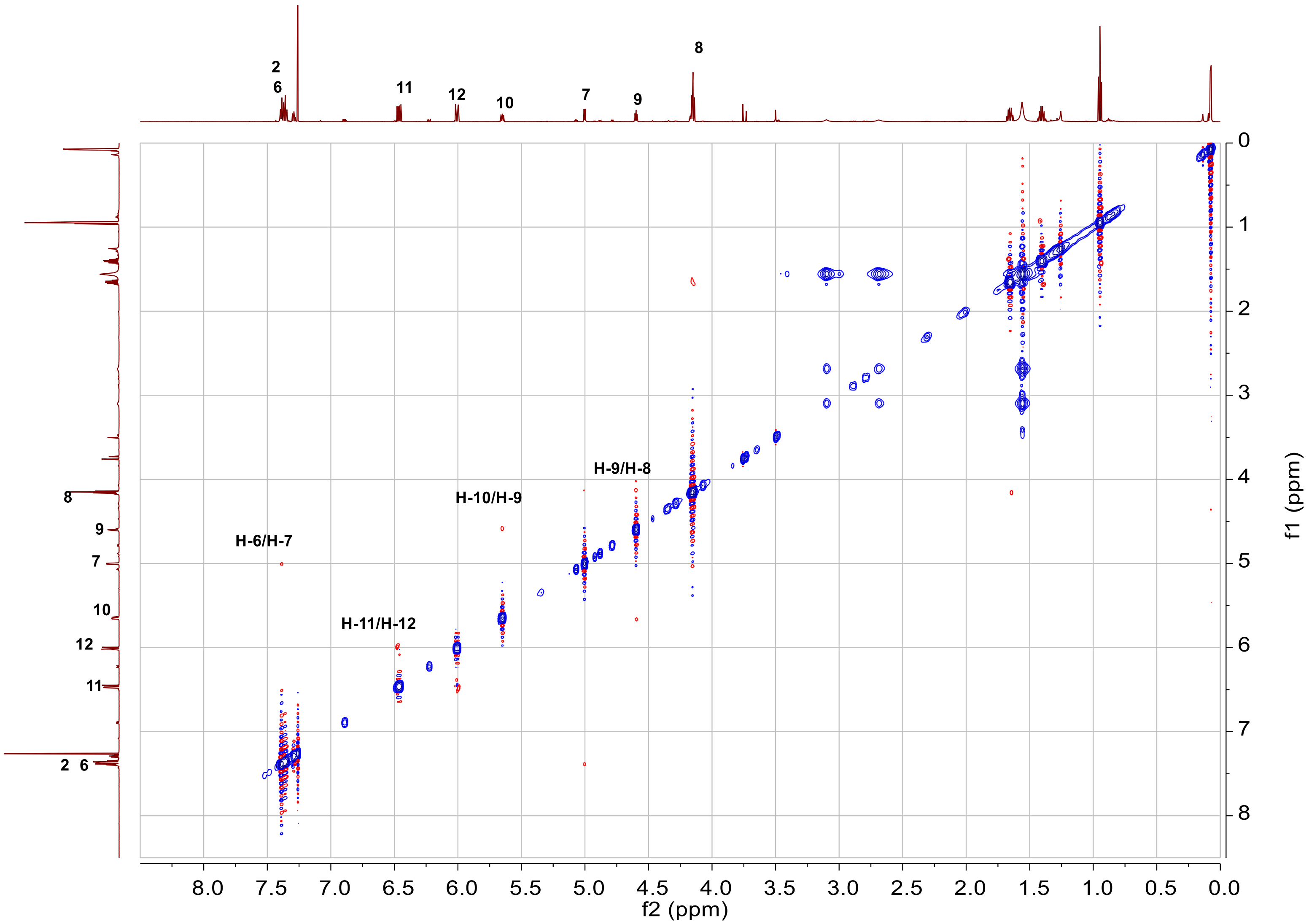

Figure S9. HRESIMS spectrum of **1**

Mass Spectrum SmartFormula Report

Analysis Info

Analysis Name	D:\Data\g4\GPD_000002.d	4/27/2020 3:31:38 PM
Method	broadband first signal	Operator: YU HSIAO-CHING
Sample Name	GP-D	Instrument: BRUKER FT-MS solariX
Comment	ESI Positive	

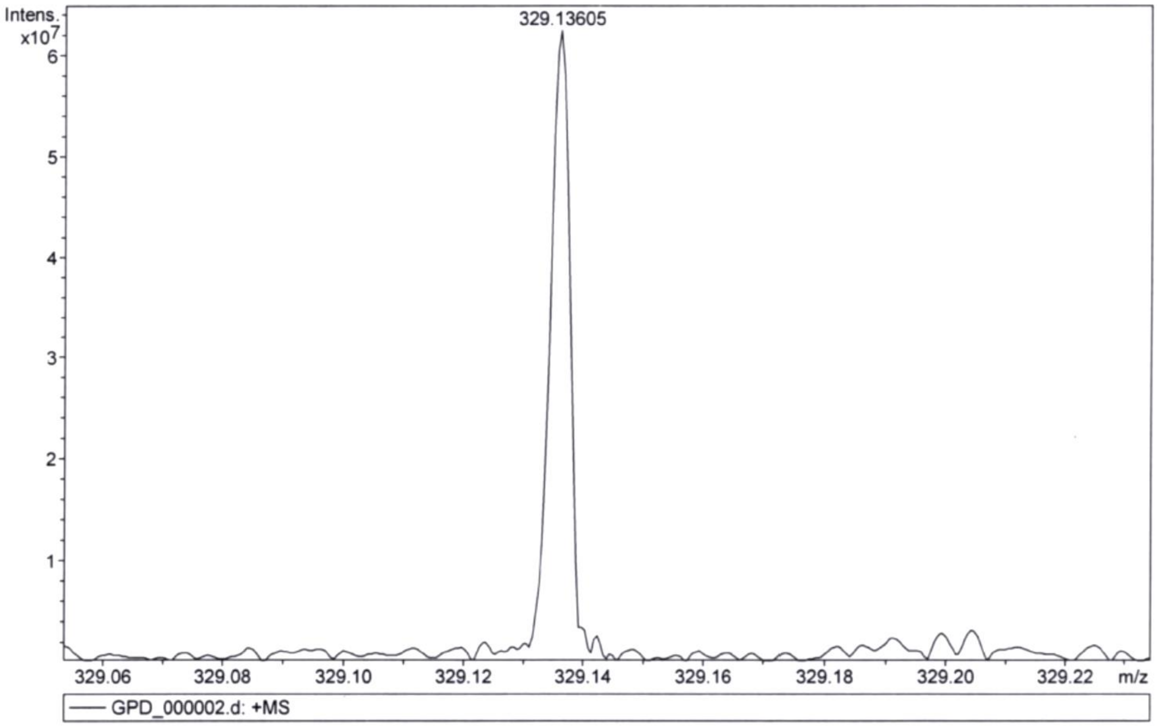

Meas. m/z	#	Formula	Score	m/z	err [mDa]	err [ppm]	mSigma	rdb	e⁻ Conf	N-Rule
329.13605	1	C 17 H 22 Na O 5	100.00	329.13594	-0.10	-0.32	16.4	6.5	even	ok

Figure S10. UV spectrum of **1**

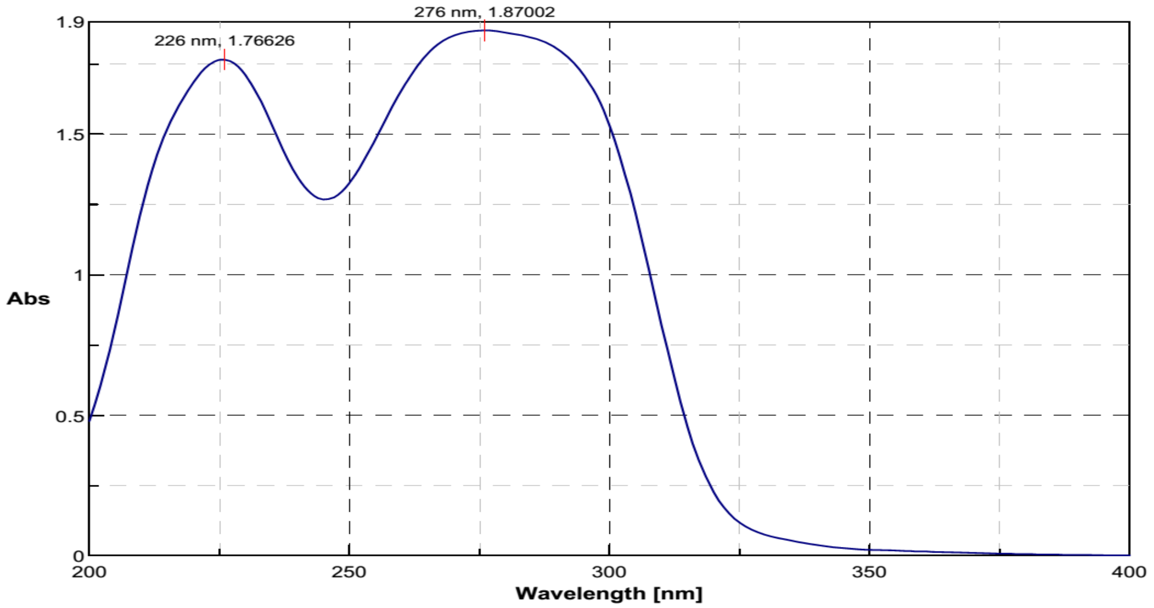

Figure S11. IR spectrum of **1**

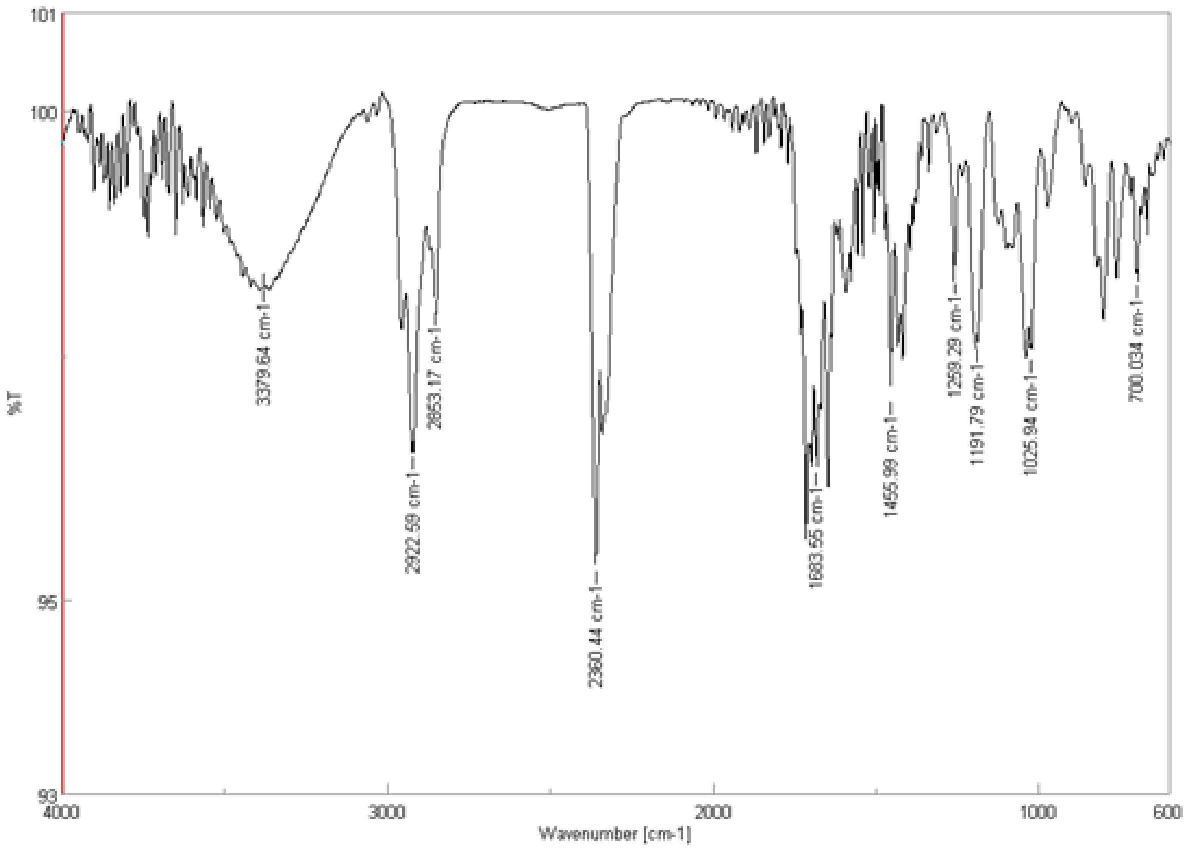

Figure S12. ^{1}H NMR spectrum of **2** ($CDCl_3$, 700 MHz)

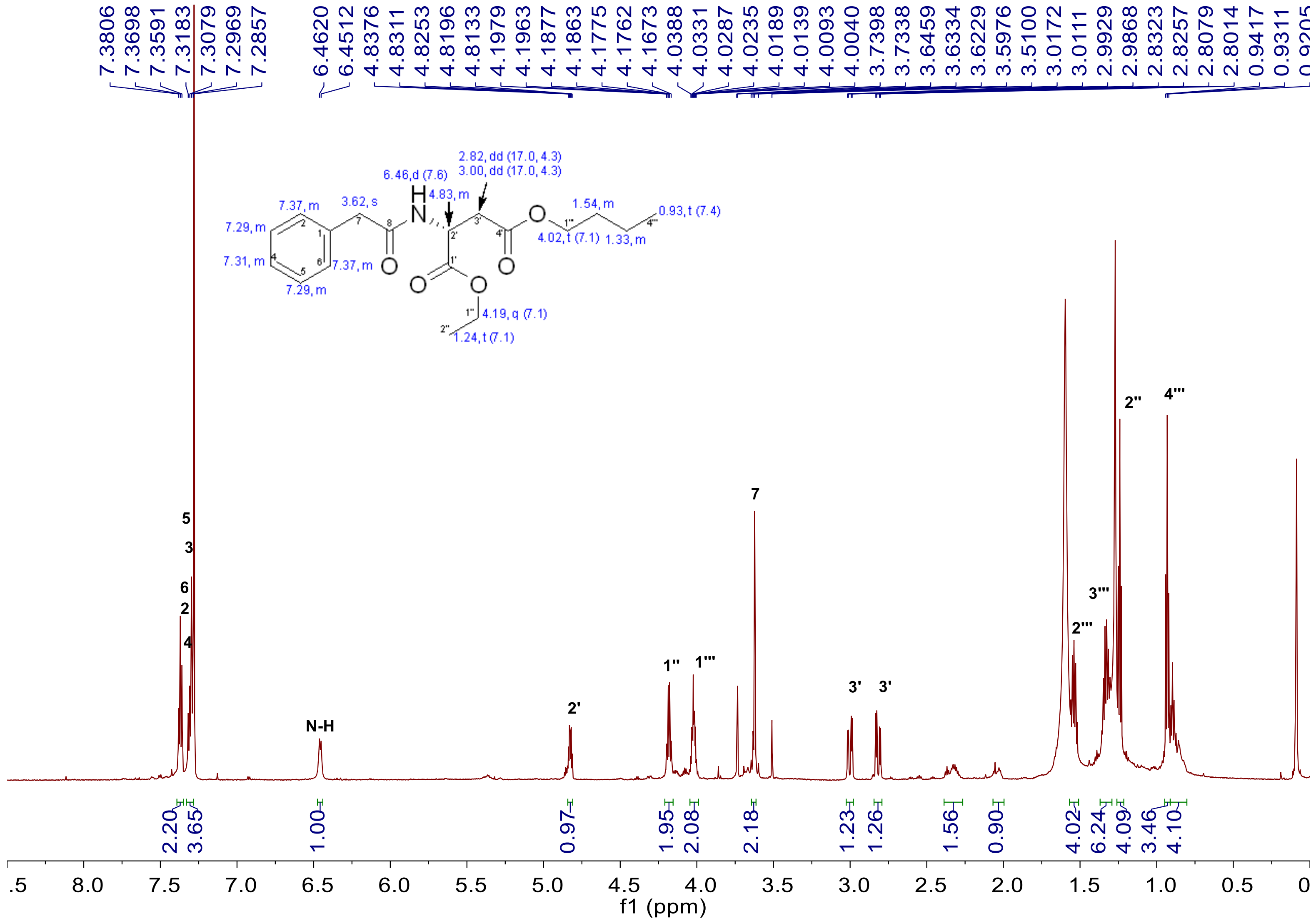

Figure S13. ^{13}C NMR and DEPT spectra of **2** ($CDCl_3$, 175 MHz)

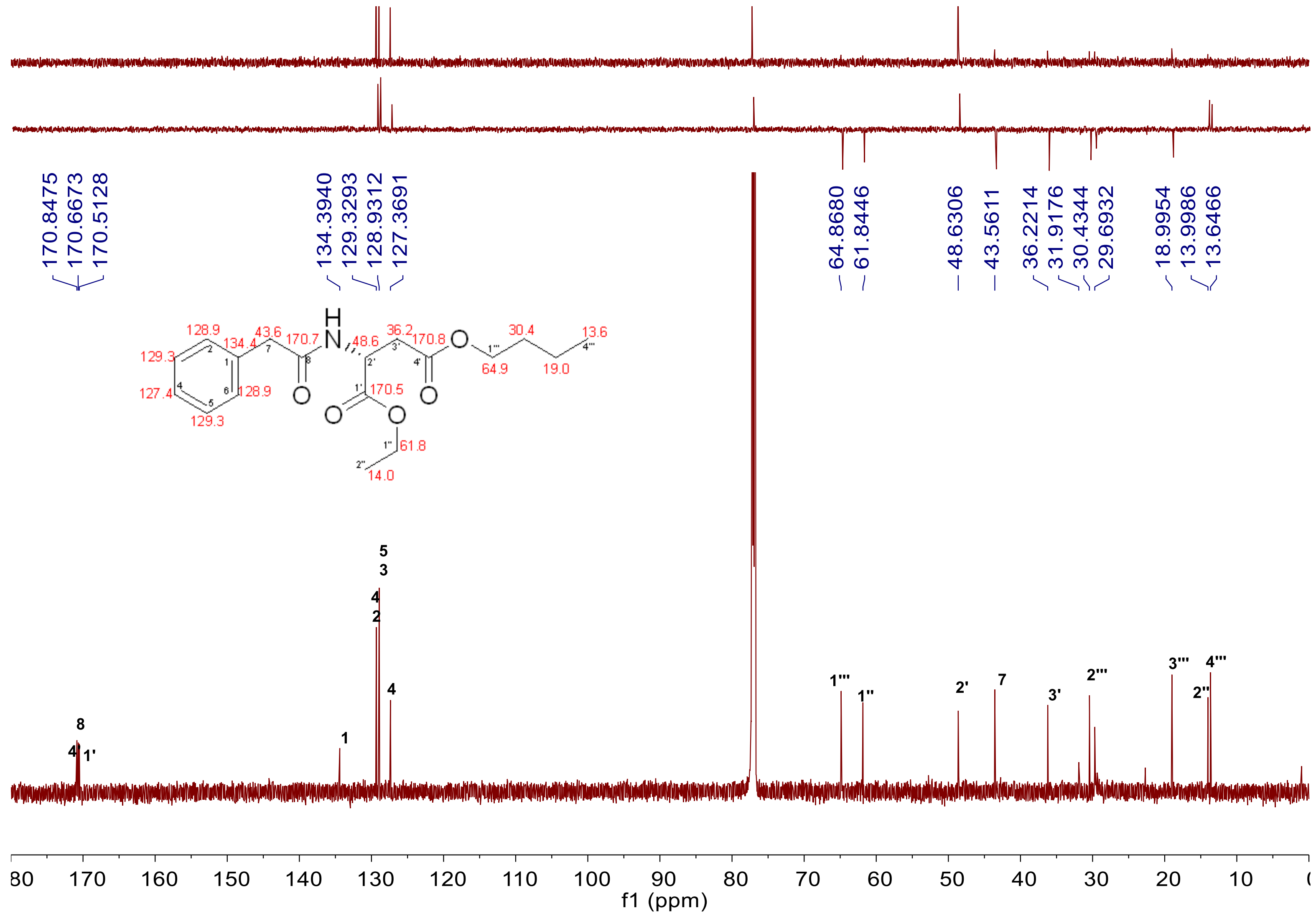

Figure S14. COSY spectrum of **2**

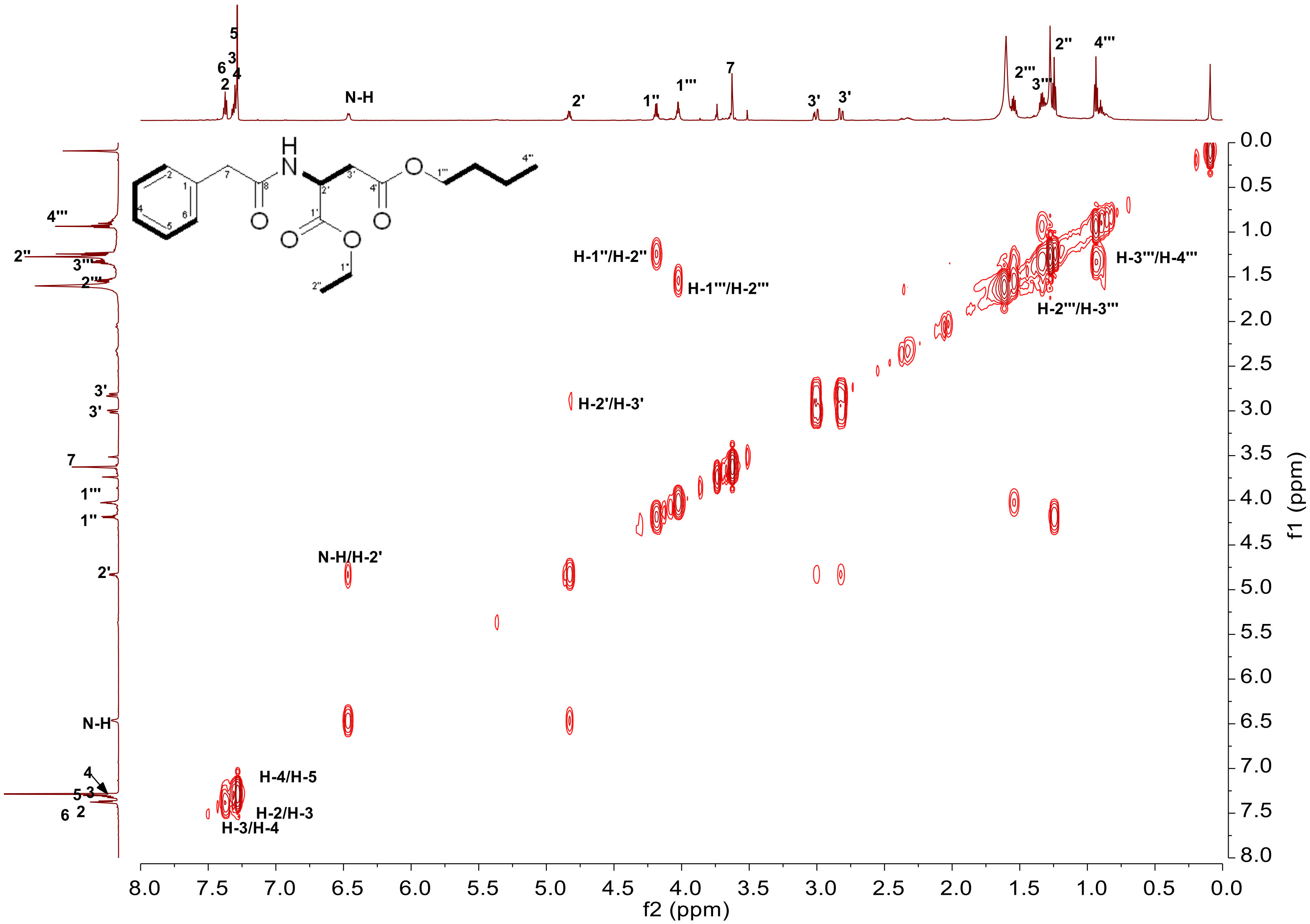

Figure S15. HSQC spectrum of **2**

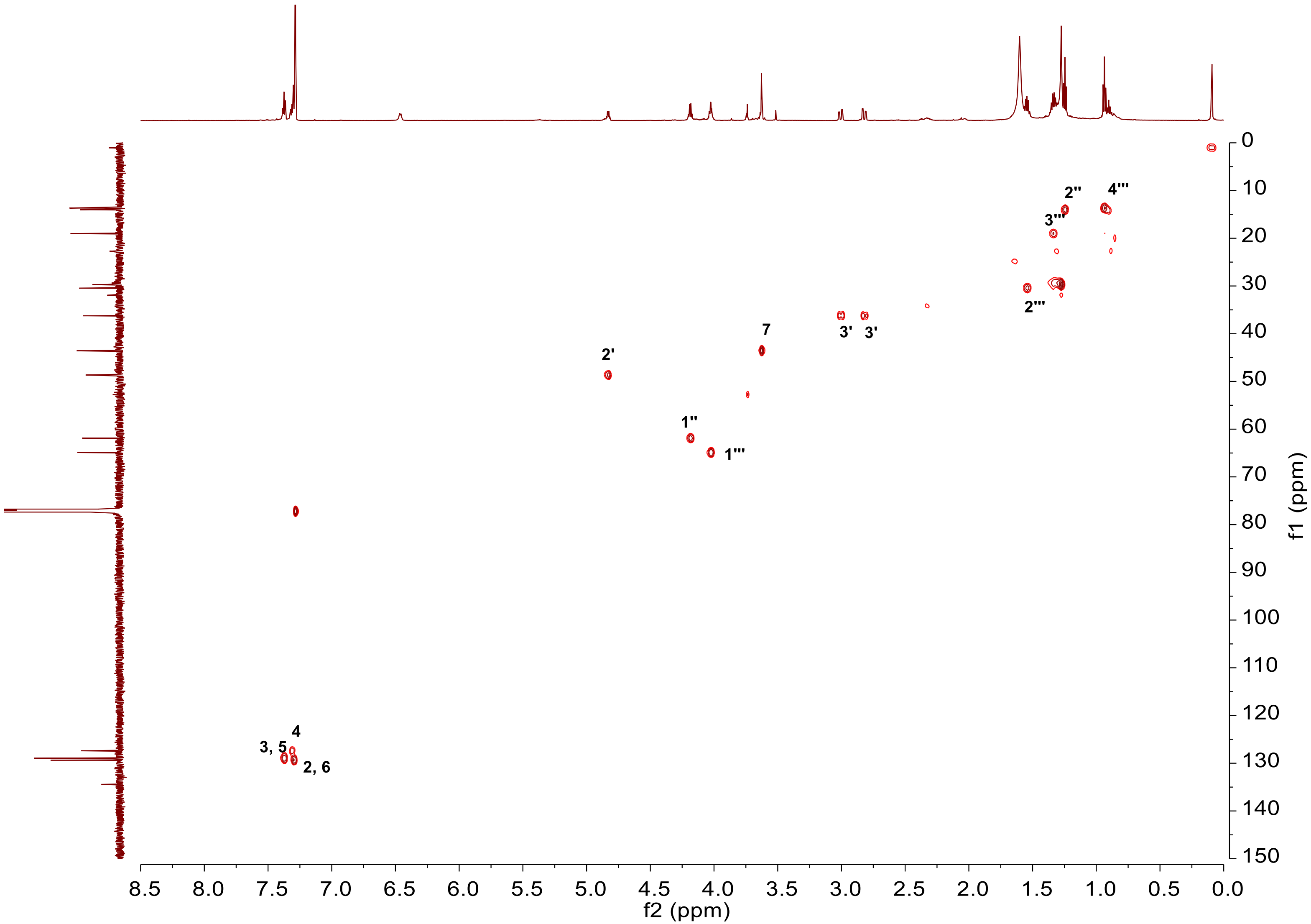

Figure S16. HMBC spectrum of **2**

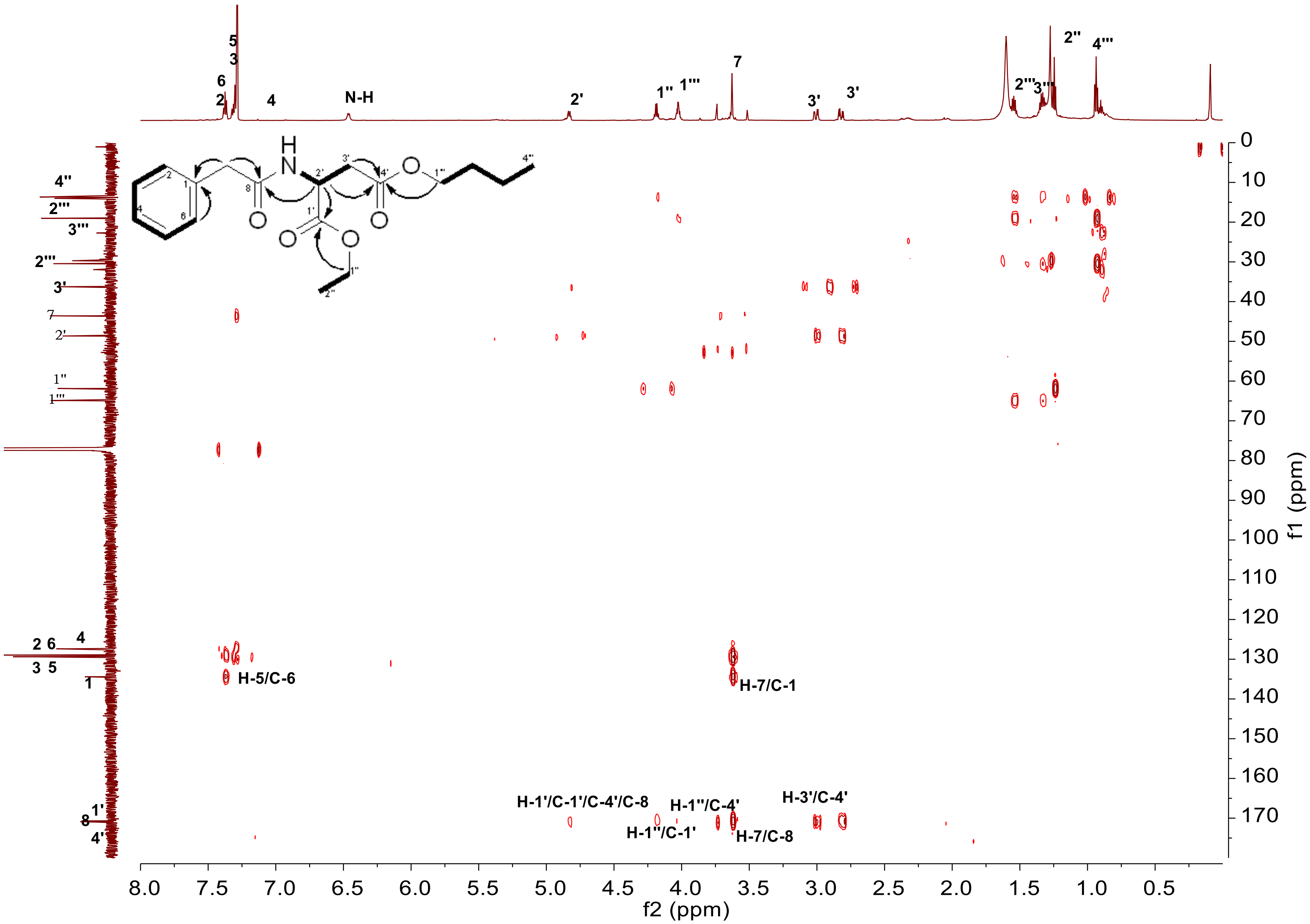

Figure S17. HRESIMS spectrum of **2**

Mass Spectrum SmartFormula Report

Analysis Info

Analysis Name	D:\Data\g4\GPE_000002.d	4/27/2020 3:36:49 PM
Method	broadband first signal	Operator: YU HSIAO-CHING
Sample Name	GP-E	Instrument: BRUKER FT-MS solariX
Comment	ESI Positive	

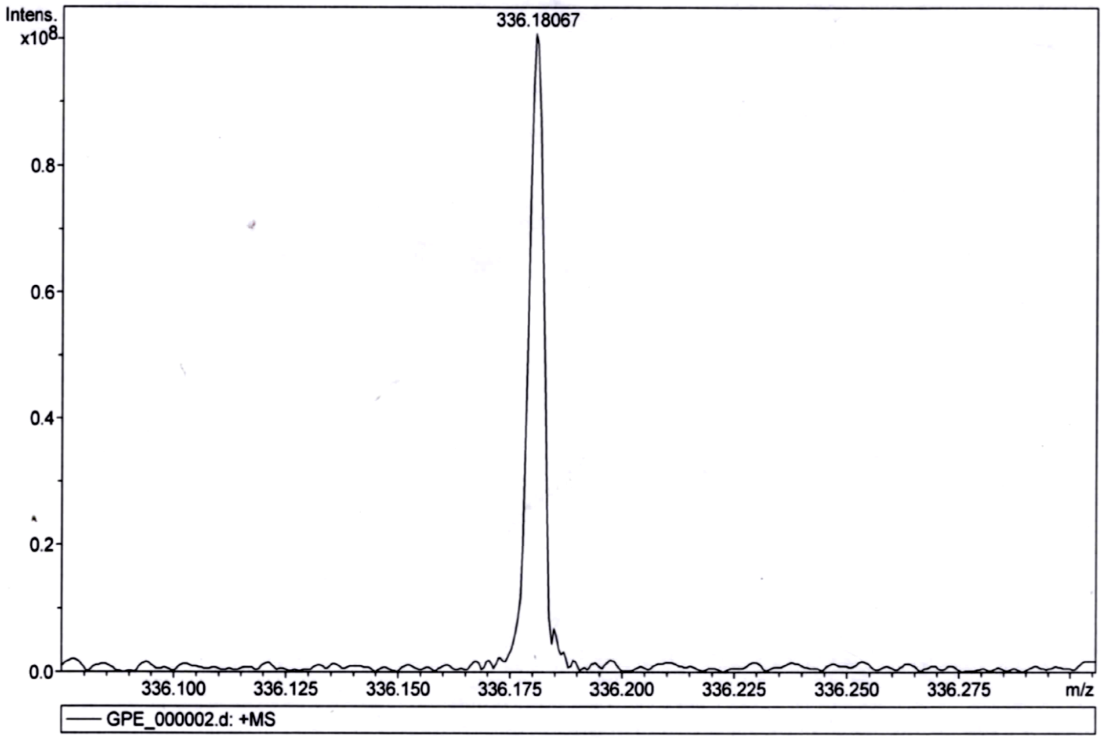

Meas. m/z	#	Formula	Score	m/z	err [mDa]	err [ppm]	mSigma	rdb	e⁻ Conf	N-Rule
336.18067	1	C 18 H 26 N O 5	100.00	336.18055	-0.12	-0.35	3.5	6.5	even	ok

Figure S18. UV spectrum of **2**

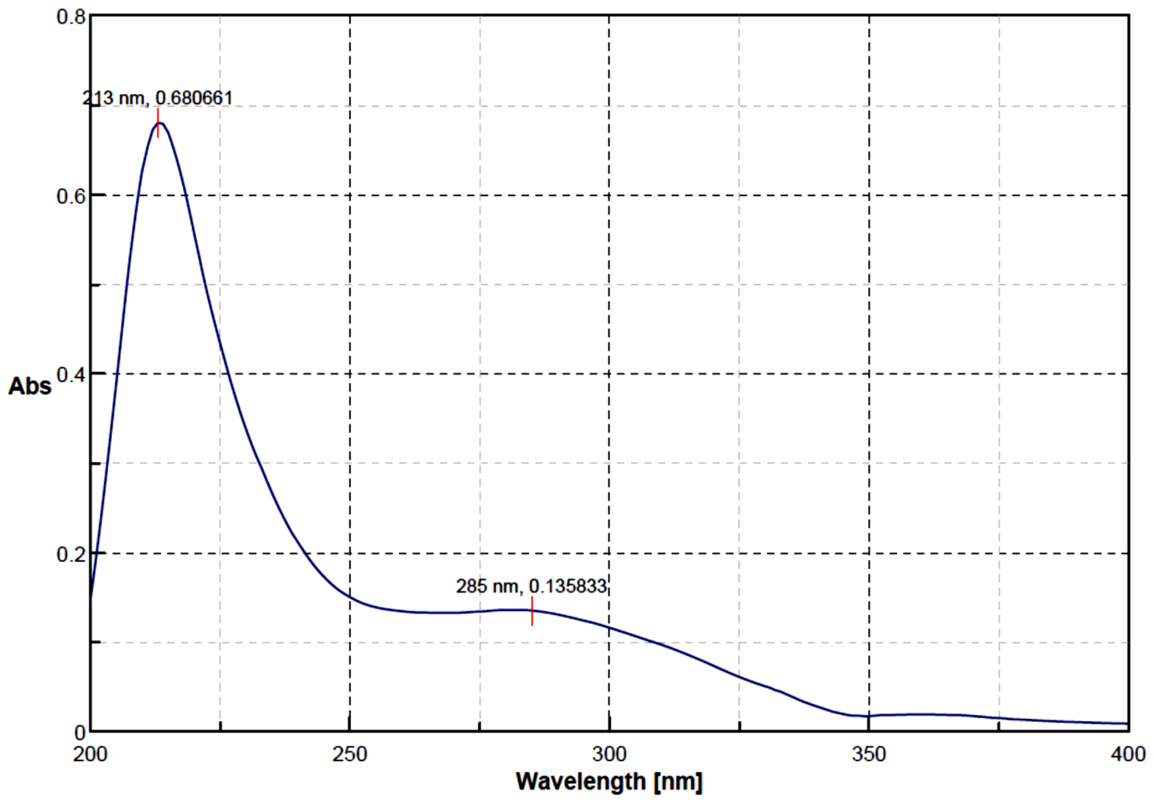

Figure S19. IR spectrum of **2**

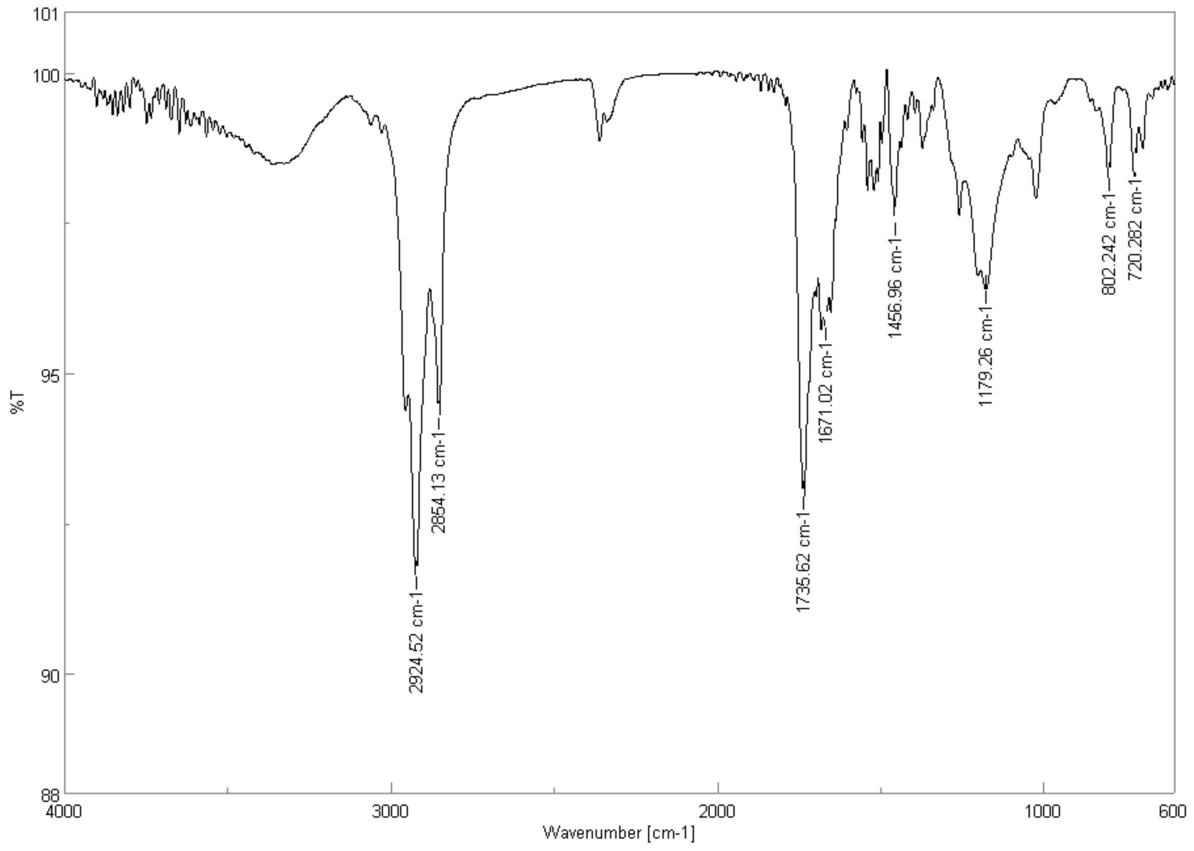

Figure S20. ^{1}H NMR spectrum of **3** ($CDCl_3$, 600 MHz)

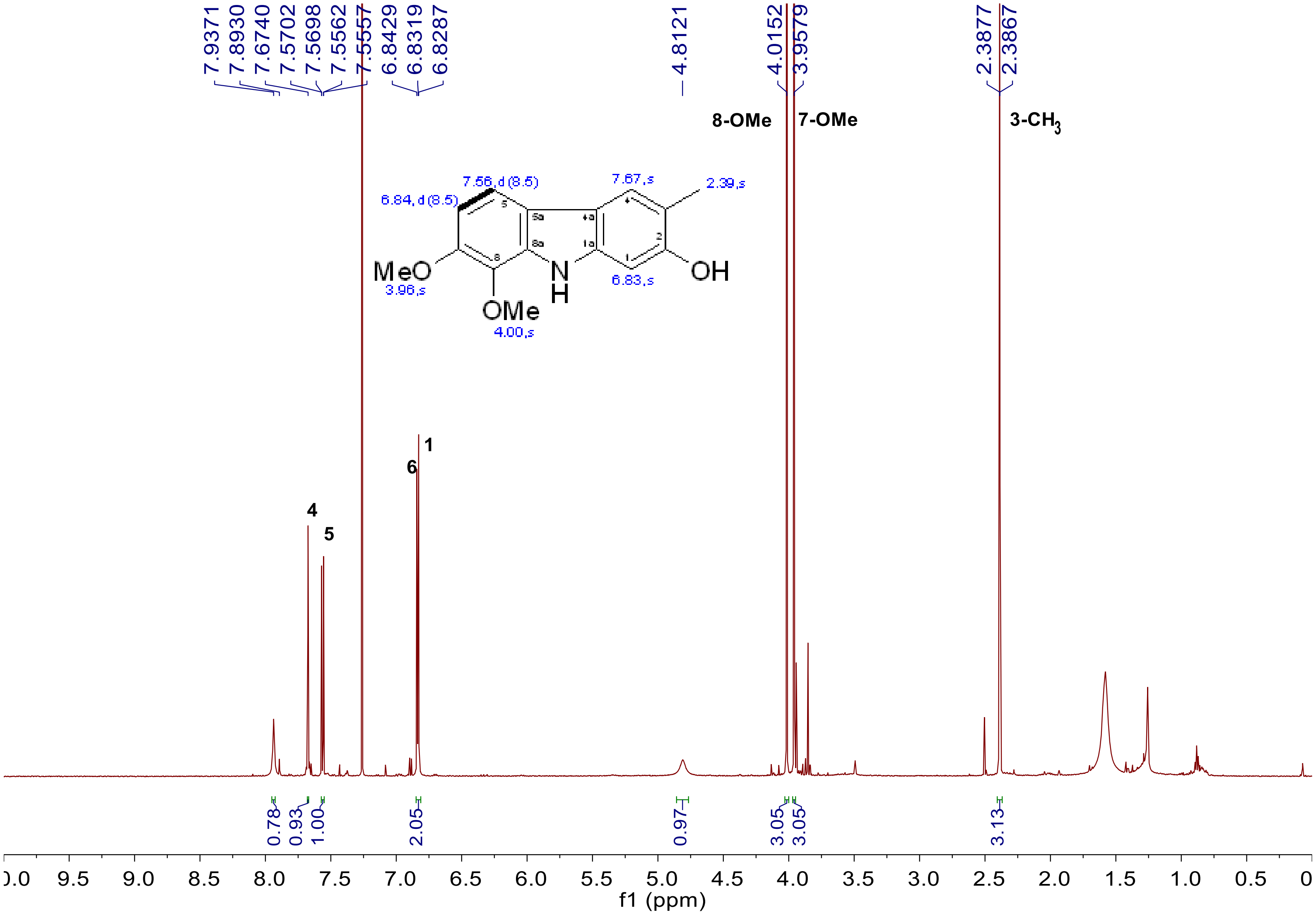

Figure S21. ^{13}C NMR and DEPT spectra of **3** ($CDCl_3$, 150 MHz)

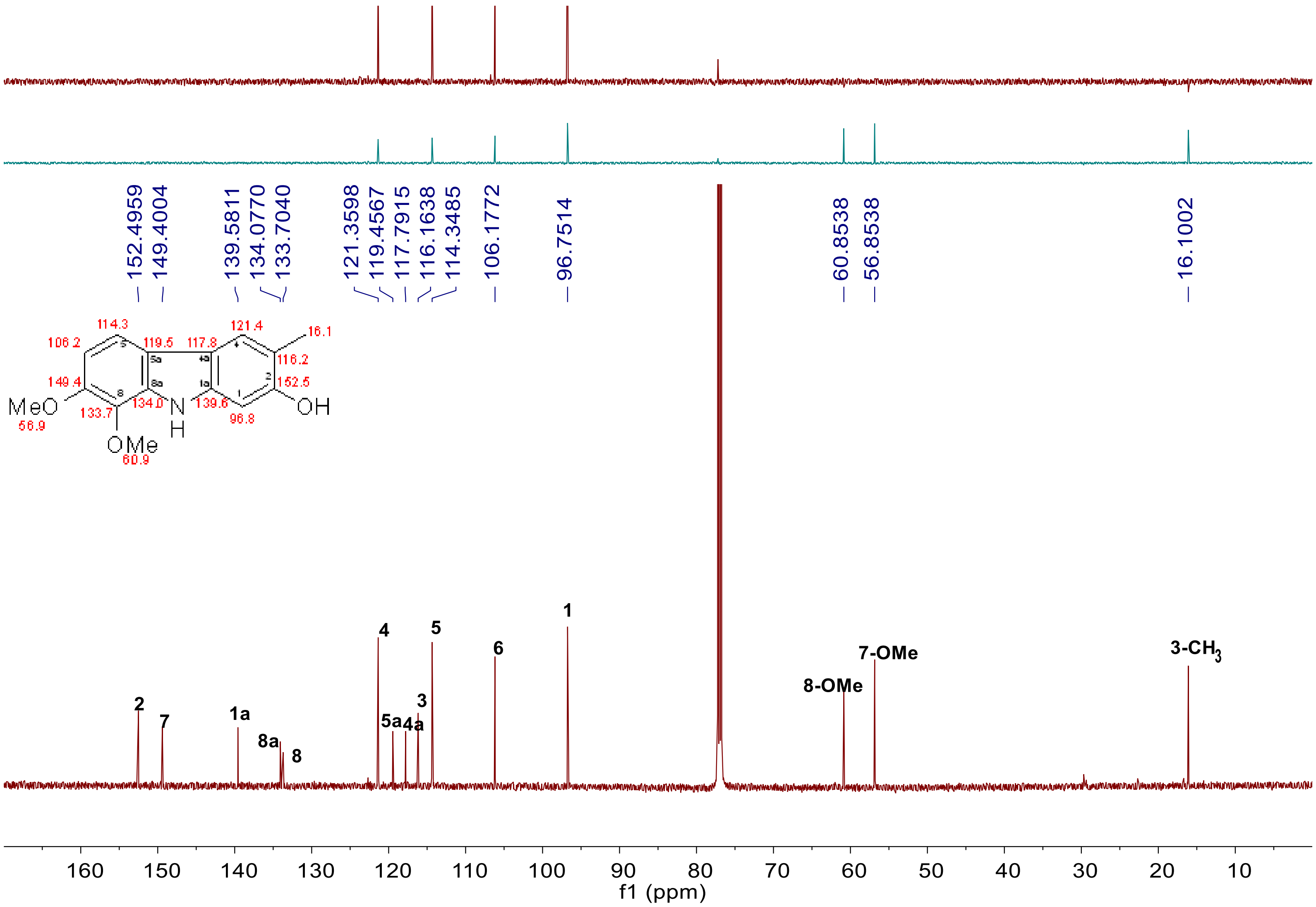

Figure S22. COSY spectrum of **3**

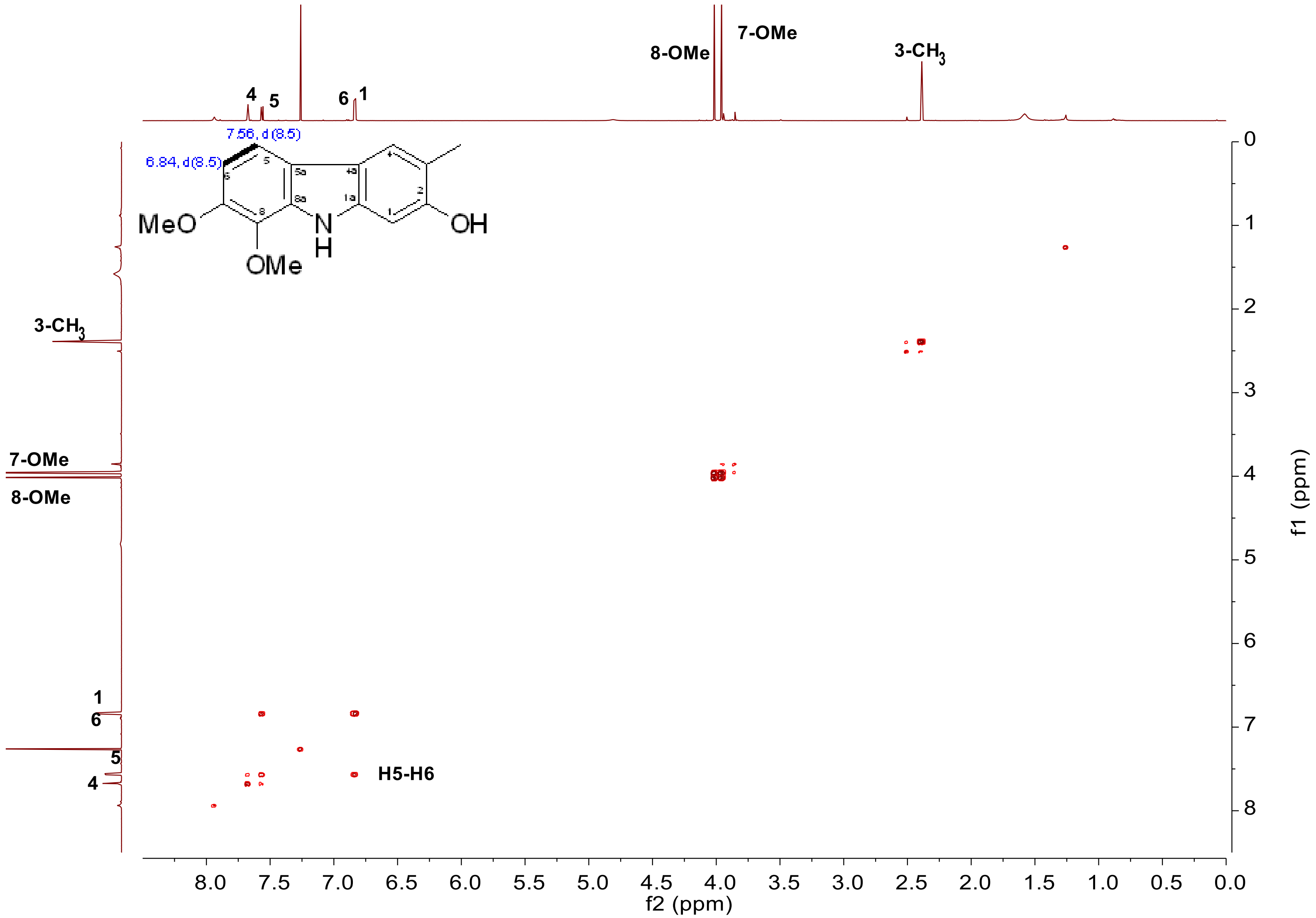

Figure S23. HSQC spectrum of **3**

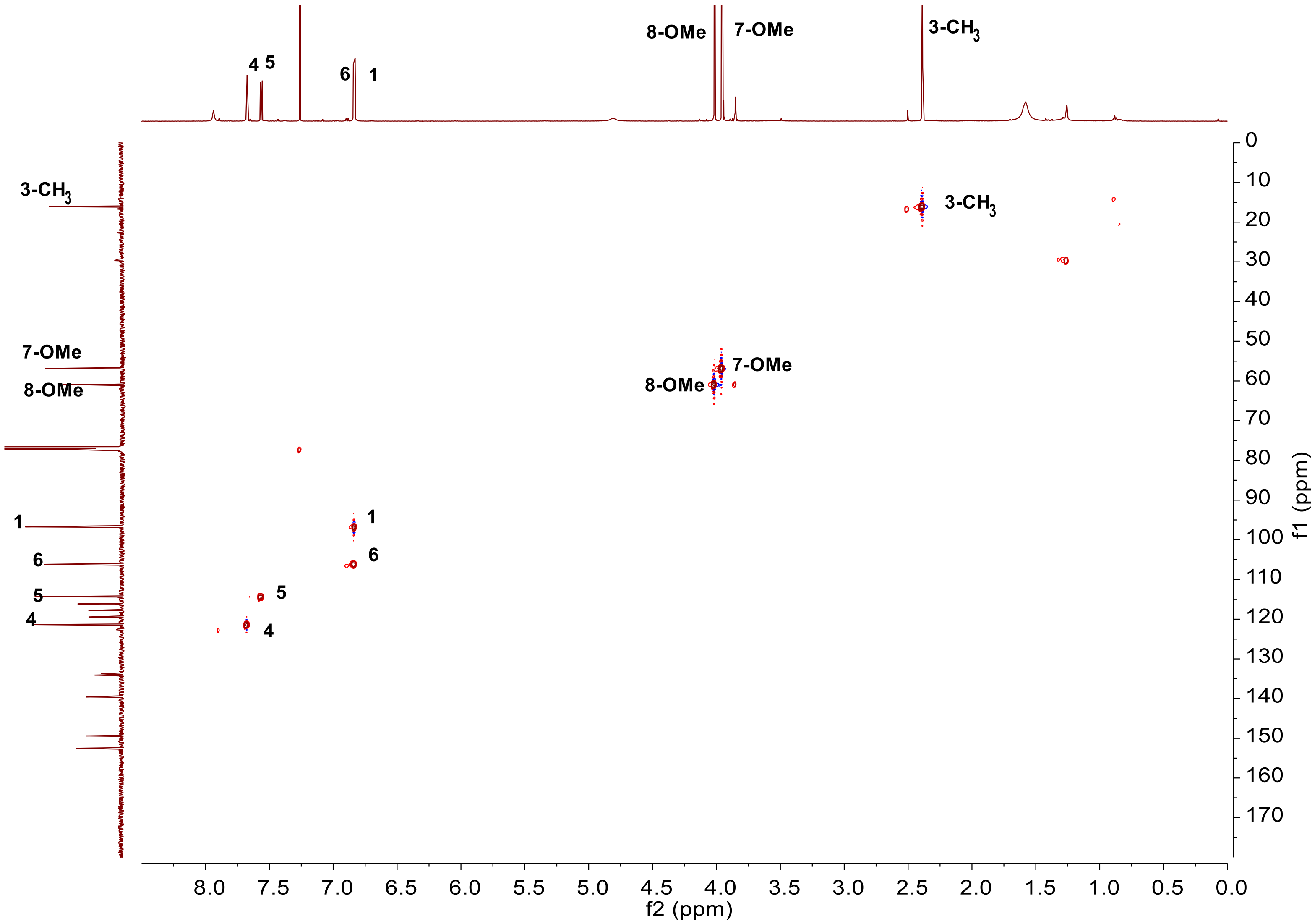

Figure S24. HMBC spectrum of **3**

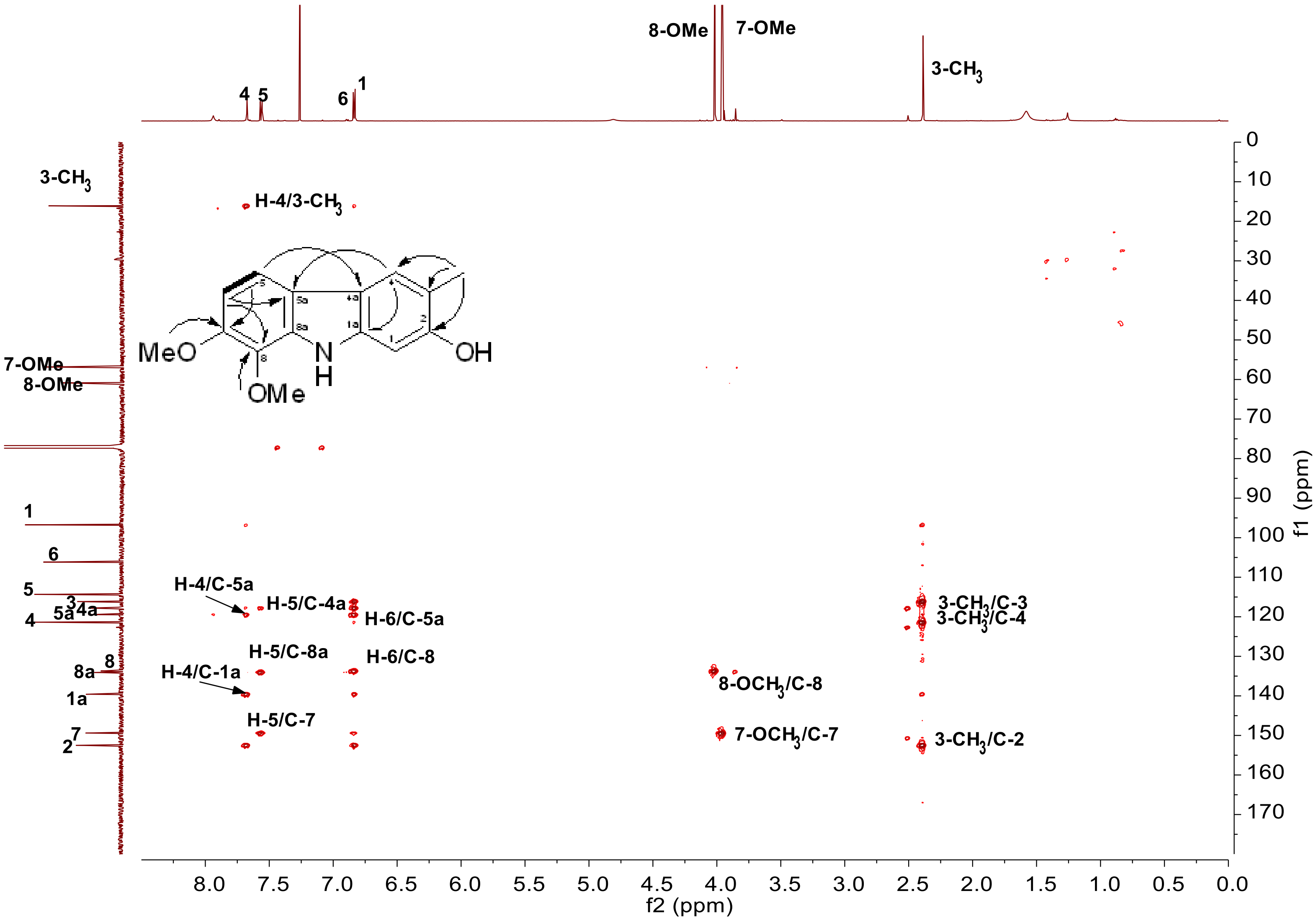

Figure S25. HRESIMS spectrum of **3**

Mass Spectrum SmartFormula Report

Analysis Info

Analysis Name	D:\Data\g4\GPF_000004.d	8/24/2020 4:51:35 PM
Method	broadband first signal	Operator: YU HSIAO-CHING
Sample Name	GP-F	Instrument: BRUKER FT-MS solariX
Comment	ESI Positive	

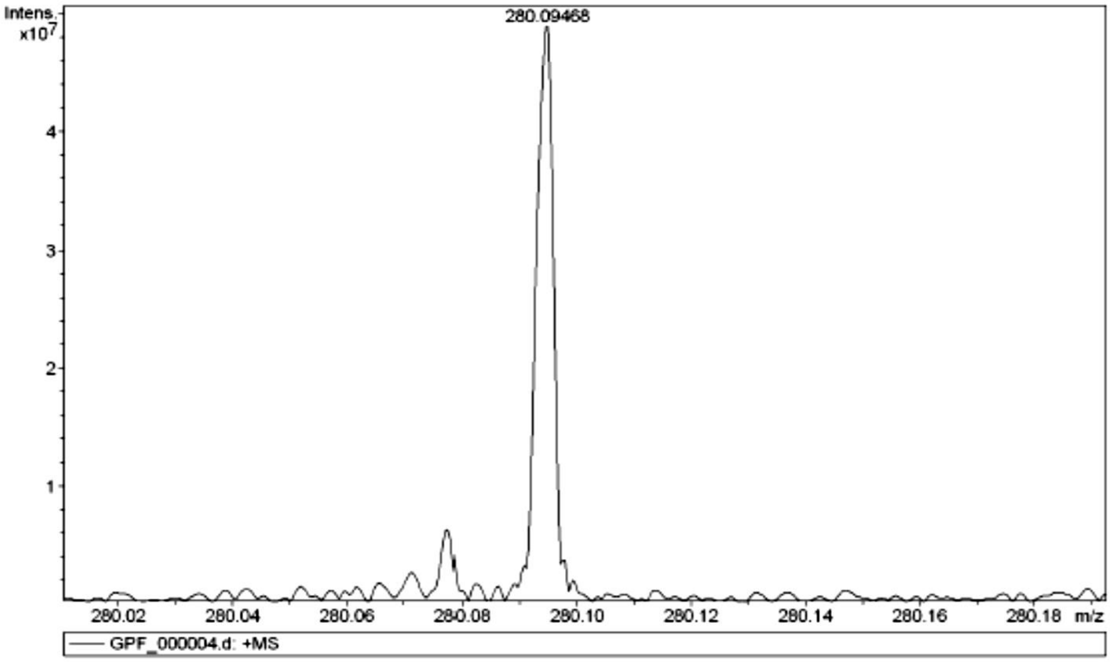

Meas. m/z	#	Formula	Score	m/z	err [mDa]	err [ppm]	mSigma	rdb	e¯ Conf	N-Rule
280.09468	1	C 15 H 15 N Na O 3	100.00	280.09441	-0.27	-0.95	13.1	8.5	even	ok

Figure S26. UV spectrum of **3**

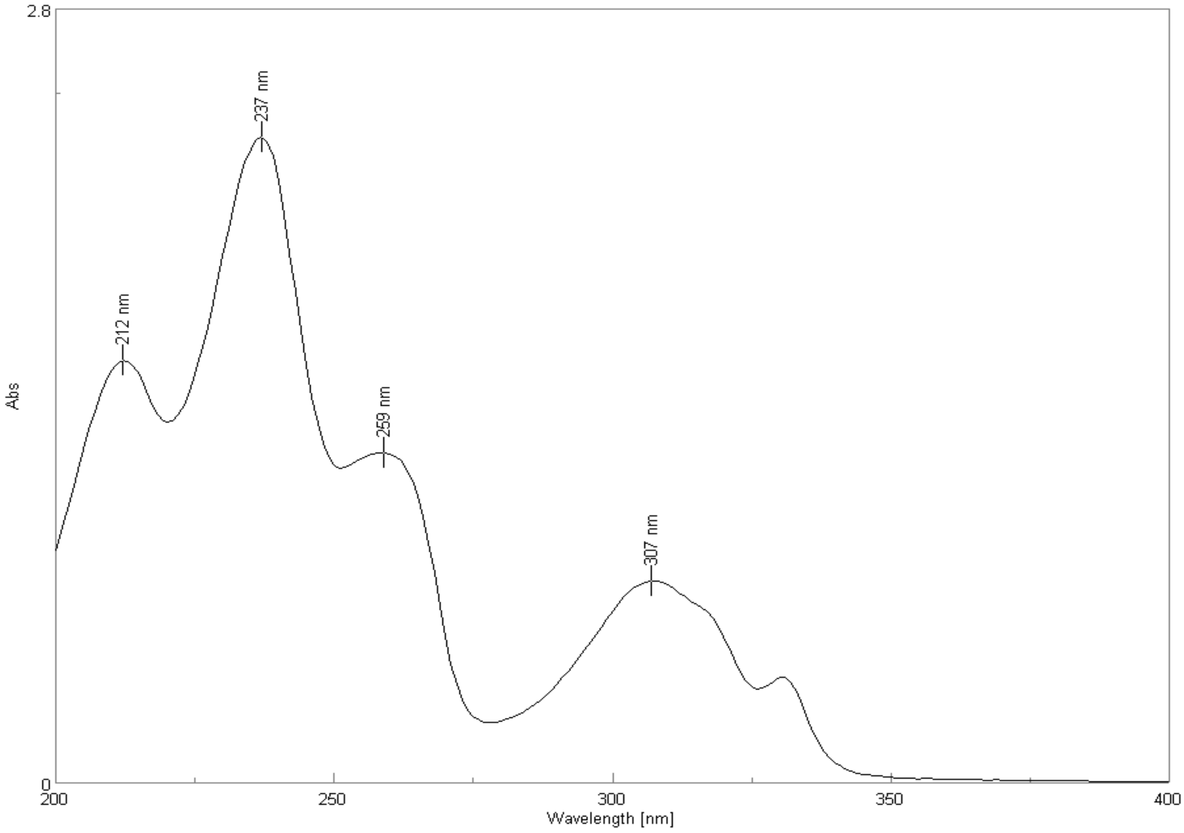

Figure S27. IR spectrum of **3**

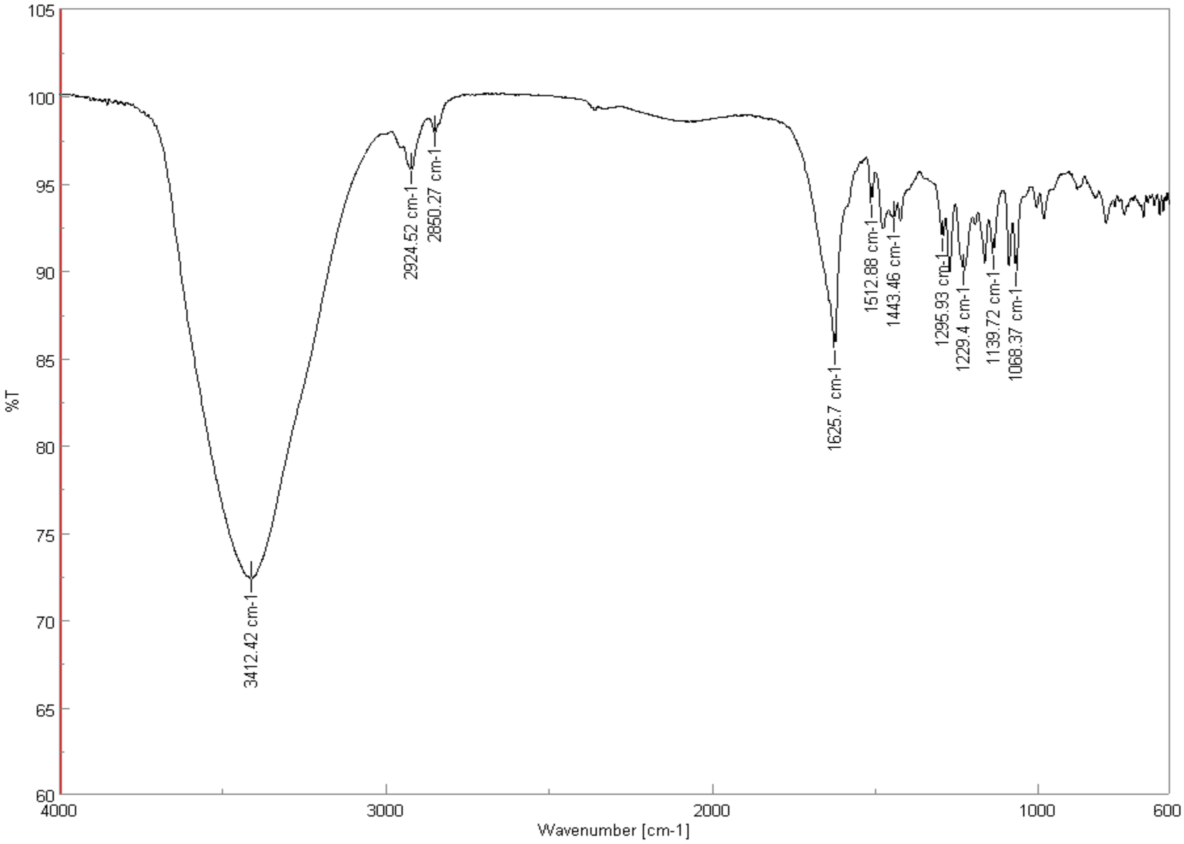

Supplement: Supplementary file 1 [file pharmaceuticals-15-01543-s001.zip › pharmaceuticals-2093381-supplementary.pdf]
